# Revealing the Diagnostic Potential of Three RHO GTPase‐Related Genes in Osteoarthritis Based on Single‐Cell RNA Sequencing and GEO Dataset Analysis

**DOI:** 10.1155/ijog/7632269

**Published:** 2026-09-28

**Authors:** Guoqiang Zou, Xufeng Wang, Jing Yu

**Affiliations:** ^1^ First Clinical College of Liaoning University of Traditional Chinese Medicine, Shenyang, Liaoning Province, China; ^2^ The Affiliated Hospital of Liaoning University of Traditional Chinese Medicine, Shenyang, Liaoning Province, China, lnutcm.edu.cn

**Keywords:** biomarkers, gene set enrichment analysis, osteoarthritis, RHO GTPase, single-cell bioinformatics analysis

## Abstract

**Background:**

Osteoarthritis (OA) is becoming increasingly common, and RHO GTPase family members have been implicated in inflammatory processes associated with OA. We therefore examined the diagnostic utility of RHO GTPase‐related genes (RGPRGs) in OA.

**Methods:**

The OA expression datasets were collected from public repositories, whereas RGPRGs were assembled from published literature. In the training cohort, we first performed differential expression analysis to screen for differentially expressed genes (DEGs) between OA and control samples. RHO GTPase‐related DEGs were obtained by intersecting the DEG list with the RGPRG set, followed by feature selection with the Boruta and XGBoost algorithms. Candidate biomarkers were further evaluated by receiver operating characteristic (ROC) analysis and cross‐dataset expression validation. The selected biomarkers were used to establish and validate an artificial neural network (ANN) model, and their biological associations were examined using gene set enrichment analysis (GSEA) and immune infiltration analysis. In GSE152805, key cell populations were identified and subsequently characterized by pseudotime, cell–cell communication, pathway activity, and metabolic analyses.

**Results:**

Analysis yielded 801 DEGs, 30 candidate genes, 14 candidate biomarkers, and 3 final biomarkers. The ANN achieved an area under the curve (AUC) of 0.940 in the training set and 0.833 in the validation set. Following Benjamini–Hochberg adjustment, VAV1, STMN2, and RND1 were associated with 34, 7, and 31 significant KEGG pathways, respectively, and five pathways were common to all three (adjusted *p* < 0.05 and |*N* *E* *S*| > 1). STMN2 also showed a significant positive association with natural killer (NK) cells (correlation coefficient (cor) = 0.604, *p* < 0.01). Fibroblasts were selected as the key cell population and displayed strong communication with endothelial cells. Along the fibroblast pseudotime trajectory, RND1 expression decreased initially, increased subsequently, and declined again at later stages. Single‐cell Reactome analysis further indicated functional differences among fibroblast subpopulations involving cell interactions, immune signaling, transcriptional regulation, and metabolic processes.

**Conclusion:**

VAV1, STMN2, and RND1 emerged as reliable diagnostic biomarkers for OA and may provide molecular candidates for early detection and therapeutic investigation. The findings also extend current insight into RHO GTPase‐related processes in OA and support further evaluation of biomarker‐based diagnostic strategies.

## 1. Introduction

Osteoarthritis (OA) is a prevalent chronic whole‐joint disorder [1] that features progressive articular cartilage destruction and associated chronic pain [2]. Its global incidence is increasing rapidly, with the number of patients expected to double by 2040 [3, 4]. Currently, the etiology and core mechanisms of OA are not fully understood [5], and clinical interventions primarily rely on symptomatic treatment, which can temporarily alleviate symptoms such as pain [6] but cannot achieve a cure. When conservative treatments fail, more aggressive interventions such as intra‐articular injections or joint replacement surgery are often employed, but these methods carry risks of complications and may provide only temporary relief [7]. Thus, further elucidating the pathological mechanisms of OA and identifying new therapeutic targets and early diagnostic biomarkers are crucial for improving OA clinical management.

The RHO GTPase family represents a core component of the small GTPase superfamily and precisely regulates processes such as cytoskeletal reorganization, proliferation, apoptosis, and inflammatory factor secretion [8, 9]. Its aberrantly activated signaling pathways have been confirmed to be intimately linked to a range of degenerative and inflammatory disorders [10–13]. In the OA pathological microenvironment, chondrocyte dedifferentiation, apoptosis, and cartilage matrix degradation are central to disease progression, with matrix metalloproteinases (MMPs) playing a key role in extracellular matrix breakdown [14–17]. Members of the RHO GTPase family can not only influence the balance between extracellular matrix synthesis and degradation by regulating the chondrocyte cytoskeleton [18, 19] but also participate in synovial fibroblast activation and inflammatory factor release, thereby exacerbating joint inflammation [20, 21]. However, the diagnostic value and specific mechanisms of RHO GTPase‐related genes (RGPRGs) in OA remain unclear and require systematic elucidation through integrative bioinformatics and single‐cell approaches.

Single‐cell RNA sequencing enables gene expression profiling at single‐cell resolution, which directly mirrors the functional heterogeneity present across different cells [22], and it aids in identifying disease‐associated differential signatures and deciphering their molecular mechanisms [23]. Integrating single‐cell expression information with functional gene sets such as RGPRGs provides a new approach for precisely identifying key cellular targets in OA.

This study aimed to integrate bioinformatics with single‐cell sequencing. We systematically investigated RHO GTPase‐related biomarkers and key cells in OA to elucidate their biological functions and regulatory mechanisms. These findings may deepen our understanding of OA pathogenesis and provide a theoretical and empirical basis for developing diagnostic and therapeutic strategies.

## 2. Materials and Methods

### 2.1. Data Collection

OA datasets from the Gene Expression Omnibus (GEO) database (https://www.ncbi.nlm.nih.gov/geo/) included GSE55235 (GPL96 platform, 10 OA and 10 normal synovial samples) as the training set, and GSE12021 (GPL96 platform, 10 OA and 9 normal samples) as the independent external validation set. The single‐cell dataset GSE152805 (GPL20301 platform) consisted of three OA synovial tissue samples. A collection of 733 RGPRGs was additionally obtained from the literature [24] (Table S1). GEO was accessed on November 1, 2025.

### 2.2. Identification and Analysis of Candidate Genes

Differential expression between disease and control samples in the training set was assessed with limma (v3.54.0) [25], using *p* adj < 0.05 and |log_2_ *f* *o* *l* *d* *c* *h* *a* *n* *g* *e* (*F* *C*)| > 1.0 as the significance criteria [26]. The DEG list was then overlapped with RGPRGs, and ggvenn (v0.1.9) [27] was used to identify OA‐associated RHO GTPase‐related DEGs, which were defined as candidate genes.

To explore the enriched biological pathways of candidate genes, the clusterProfiler package (v4.7.1) [28] was employed to perform Gene Ontology (GO) and Kyoto Encyclopedia of Genes and Genomes (KEGG) analyses. Additionally, using the Search Tool for the Retrieval of Interacting Genes/Proteins (STRING) database, protein‐level interactions among the candidate genes were obtained, applying a confidence score threshold of 0.15.

### 2.3. Screening of OA‐Related Biomarkers by Machine Learning

Candidate genes were subjected to two complementary machine learning procedures in the training set: Boruta and Extreme Gradient Boosting (XGBoost). Boruta evaluates all relevant features by comparing each observed variable with randomly generated shadow variables, which helps retain informative genes in high‐dimensional expression data. XGBoost, by contrast, captures nonlinear effects and gene interactions while assigning feature‐importance scores according to their contribution to classification. Using both algorithms provides complementary evidence and was intended to strengthen the stability and biological relevance of biomarker selection.

Candidate genes were first processed with the Boruta package (v8.0.0) [29] for 30 iterations at *p* < 0.01 to obtain the Boruta candidate biomarkers. XGBoost was then iteratively optimized and used to rank candidate genes by feature importance. The analysis was implemented with XGBoost (v1.7.3.1) [30] using 30 iterations, a maximum depth of 6, and fivefold cross‐validation, yielding the XGBoost_candidate biomarkers. The two feature sets were intersected with ggvenn (v0.1.9) [27] to derive the final set of candidate biomarkers.

Candidate biomarkers then underwent ROC analysis and expression validation; pROC (v1.18.0) [31] generated ROC curves for training and validation sets with AUCs, and those with AUC > 0.7 were kept as key candidates showing acceptable diagnostic performance [32]. Expression levels were then compared between disease and control groups across both datasets via the Wilcoxon rank‐sum test, and biomarkers with significant differential expression and consistent directional changes in both training and validation sets were selected as the final biomarkers.

### 2.4. Construction and Validation of an ANN

To assess whether the selected biomarkers could discriminate OA from control samples, an ANN was trained on the GSE55235 dataset with the neuralnet package (v1.44.2) [33]. VAV1, STMN2, and RND1 expression values were independently transformed to the [0,1] interval by min–max normalization before model fitting. The network used a 3–3–2 structure: three input neurons representing VAV1, STMN2, and RND1, one hidden layer containing three neurons, and two output neurons corresponding to OA and control classes. Logistic sigmoid activation was specified for both the hidden and output layers (linear.output = FALSE). Cross‐entropy served as the error function (err.fct = “ce”), and weights were optimized by resilient backpropagation with weight backtracking (rprop+). The convergence threshold was 0.5, the maximum number of training steps was 1 × 10^5^, and the random seed was 22. Each sample was assigned to the class with the larger output‐neuron value, and the OA output score was used for ROC analysis. Model performance, evaluated in the GSE55235 training set and independently tested in GSE12021 using pROC (v1.18.0) [31], was considered favorable for OA diagnosis when AUC exceeded 0.7.

### 2.5. Gene Set Enrichment Analysis (GSEA)

GSEA was conducted in the training set to characterize biological processes related to each biomarker. We computed Spearman correlations between each biomarker and all other genes using psych (v2.2.9) [34], then ranked genes by descending correlation coefficient to produce a gene list for each biomarker. The c2.cp.kegg.v7.5.1.symbols.gmt collection from the Molecular Signatures Database (MSigDB) was used as the reference gene set, and enrichment analysis was performed with clusterProfiler (v4.7.1) [28]. *p* values were adjusted using the Benjamini–Hochberg method; pathways with BH‐adjusted *p* < 0.05 and |*n* *o* *r* *m* *a* *l* *i* *z* *e* *d* *e* *n* *r* *i* *c* *h* *m* *e* *n* *t* *s* *c* *o* *r* *e* (*N* *E* *S*)| > 1 were deemed significant.

### 2.6. Immune Infiltration Analysis

Immune infiltration within the training set was estimated via ssGSEA [35], producing abundance scores for 28 immune cell types [36]. The Wilcoxon rank‐sum test compared disease and control scores, and cell types showing *p* < 0.05 were defined as differentially infiltrated immune cells (DIICs). We then examined associations between DIICs and biomarkers via Spearman correlation using psych (v2.2.9) [34], with *p* < 0.05 and |*c* *o* *r* *r* *e* *l* *a* *t* *i* *o* *n* *c* *o* *e* *f* *f* *i* *c* *i* *e* *n* *t* (*c* *o* *r*)| > 0.3 as cutoffs [37].

### 2.7. Construction of the Regulatory Network of Biomarkers

To identify transcription factors (TFs) with potential regulatory effects on the biomarkers and microRNAs (miRNAs) that may interact with them, predictions were performed using the Network Analyst online platform (https://www.networkanalyst.ca/). In addition, to map interactions between the biomarkers and their functionally related genes, the Gene Multiple Association Network Integration Algorithm (GeneMANIA) database was employed to predict genes functionally associated with the biomarkers, uncover their potential biological functions, and establish a corresponding gene interaction network.

### 2.8. Drug Prediction and Molecular Docking

To predict potential drugs targeting the identified biomarkers for OA therapy, biomarkers were imported into the Drug Signatures Database (DSigDB) for drug prediction. Furthermore, molecular docking was conducted using the Complete Blind‐Dock (CB‐dock) tool to assess the binding of the top‐predicted drug to its biomarker‐encoded protein target. Protein three‐dimensional (3D) structures were sourced from the Protein Data Bank (PDB) (http://www.rcsb.org) and UniProt (https://www.uniprot.org/), and the drug structure from PubChem (https://pubchem.ncbi.nlm.nih.gov/). The simulations yielded the corresponding binding energies. A lower binding energy value indicated better docking efficiency; when the binding energy was less than −5 kcal/mol, an ideal binding mode and strong affinity were considered to exist between the potential drugs and the proteins encoded by biomarkers.

### 2.9. Single‐Cell Data Processing and Clustering

Single‐cell data were processed in Seurat (v5.0.1) to obtain high‐quality cell populations [38]. Genes present in fewer than 3 cells were removed, and only cells with at least 200 detected genes were kept. Quality filtering retained cells meeting 200–6000 genes, mitochondrial proportion < 10%, and 200–50,000 UMIs. After log‐normalization, variable gene selection, and scaling, PCA was applied to variable genes; JackStraw and ElbowPlot determined significant PCs (*p* < 0.05). Batch effects were subsequently corrected with Harmony (v1.2.4) [39]. Clusters were generated with the FindClusters function at a resolution of 0.4. UMAP was then applied to the PCA‐processed data for additional dimensionality reduction, and DimPlot was used to display cell‐type partitions in low‐dimensional space.

For cluster annotation, FindAllMarkers was used to identify DEGs for each cluster with min.pct = 0.25 and logfc.threshold = 1. Canonical marker sets representing different cell types were assembled from published literature [40] and the CellMarker database (http://xteam.xbio.top/CellMarker/). Cell identities were then assigned based on the expression patterns of known marker genes across clusters.

### 2.10. Identification of Key Cells

Key cells were selected in a stepwise manner. The relative proportion of every annotated cell type was first calculated in OA samples, after which biomarker expression and distribution were examined across cell types. Cell populations showing both high abundance and biomarker expression in OA samples were defined as key cells for downstream analyses.

### 2.11. Cell Communication Analysis

To clarify intercellular interaction dynamics and compare pathway‐level information flow, cellular communication was analyzed, and the intercellular ligand‐receptor communication network was dissected using the CellChat package (v1.6.1) [41] based on single‐cell data.

### 2.12. Cell Trajectory Analysis

Pseudotime analysis was used to reconstruct the differentiation trajectory of the key cells. The key‐cell population first underwent secondary dimensionality reduction and clustering, after which each subpopulation was analyzed with Monocle (v2.26.0) [42]. Pseudotemporal ordering was then generated with the orderCells function, yielding the inferred differentiation trajectory across key‐cell subpopulations.

Biomarker expression was further examined along the inferred differentiation sequence to characterize dynamic changes across key‐cell states in the single‐cell data.

### 2.13. Single‐Cell Reactome Pathway Activity Analysis of Key Cell Subpopulations

Pathway activity differences among key‐cell subpopulations were assessed from the single‐cell RNA sequencing data with ReactomeGSA (v1.12.0) [43]. After quality control, retained cells had 200–6000 detected genes, < 50,000 total RNA counts, and < 10% mitochondrial genes; genes present in ≥ 3 cells were kept. The annotated Seurat object was analyzed with the analyse_sc_clusters function. Reactome pathway activity was estimated using a GSVA‐like rank‐based procedure and summarized as mean scores for each cell subpopulation. Importantly, ReactomeGSA/GSVA used the expression matrix of the identified key‐cell subpopulations directly rather than a preranked gene list or a list of differentially expressed genes derived from the bulk transcriptomic analysis. Accordingly, neither a gene‐ranking metric nor a permutation test was used in this analysis.

The human Reactome pathway collection implemented in ReactomeGSA (v1.12.0) was used. Pathways containing fewer than 15 or more than 500 genes were excluded. For each remaining pathway, variability across cell types was quantified as the difference between its maximum and minimum enrichment scores, and the 10 pathways with the largest ranges were selected for visualization.

To reduce redundancy caused by the hierarchical structure and shared gene members of Reactome pathways, a gene‐overlap‐based pathway collapsing analysis was further performed. The Reactome pathways identified by ReactomeGSA were matched to the CP: REACTOME gene sets obtained through the msigdbr package. Pairwise pathway similarity was calculated using the Jaccard index, and pathways with a Jaccard similarity ≥ 0.30 were grouped using a greedy clustering procedure. Within each cluster, the pathway showing the largest range of activity scores across fibroblast subpopulations, calculated as the maximum score minus the minimum score, was retained as the representative pathway.

### 2.14. UCell‐Based Hallmark and KEGG Pathway Activity Analysis

To provide complementary computational validation using alternative gene set collections, pathway activity analysis was further performed for the fibroblast subpopulations identified from GSE152805. Hallmark and KEGG (CP: KEGG) gene sets were obtained from the MSigDB using the msigdbr package (v10.0.2) [44]. UCell (v2.6.2) [45] was used to calculate rank‐based pathway activity scores for individual cells. Differences in pathway activity scores among fibroblast subpopulations were subsequently evaluated using the limma package (v3.56.2) [46]. Multiple testing correction was performed using the Benjamini–Hochberg method, and pathways with an adjusted *p* value < 0.05 were considered statistically significant. Significant pathways were ranked according to the range of their activity scores across fibroblast subpopulations, calculated as the maximum score minus the minimum score, and the Top 20 pathways were visualized using heatmaps and bubble plots with pheatmap (v1.0.12) and ggplot2 (v4.0.3).

### 2.15. Evaluation of Metabolic Activity

Metabolic pathway activity in key‐cell subpopulations was evaluated in the single‐cell dataset with scMetabolism (v0.2.1) [47]. GSVA was used to derive pathway activity values, which were subsequently compared among the different key‐cell subpopulations to characterize their metabolic profiles.

### 2.16. Statistical Analysis

All statistics were performed in R (v4.2.2), using the Wilcoxon rank‐sum test for between‐group comparisons and Spearman′s test for correlations, with significance defined as two‐tailed *p* < 0.05.

## 3. Results

### 3.1. Identification and Analysis of RHO GTPase‐Related Candidate Genes in OA

Differential expression analysis identified 801 DEGs in the disease versus control comparison within the GSE55235 training set (Table S2), with 490 upregulated and 311 downregulated (*p* adj < 0.05 and |log_2_ *f* *o* *l* *d* *c* *h* *a* *n* *g* *e* (*F* *C*)| > 1.0) (Figure 1a). The expression of the Top 5 DEGs in each direction is shown in Figure 1b. Subsequent intersection of these DEGs with the RGPRG set yielded 30 candidate genes (Figure 1c) (Table S3).

**Figure 1:**
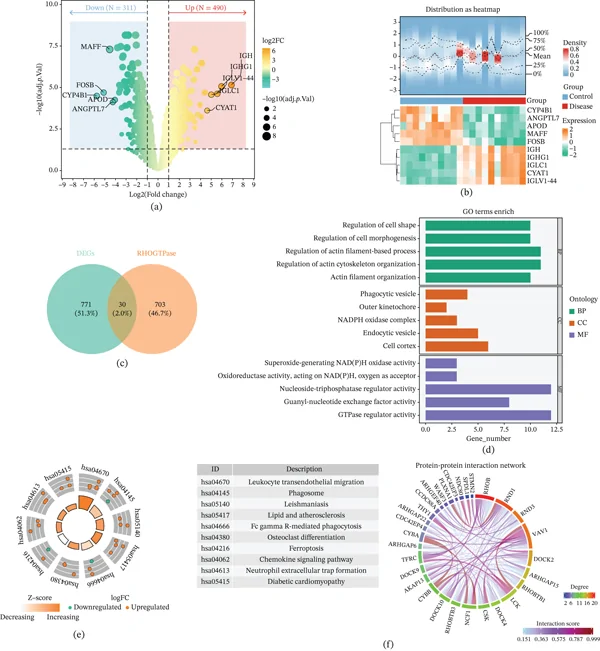
Identification of differentially expressed genes (DEGs) and candidate genes. (a) Volcano plot showing the DEGs between disease and control groups; green represents significantly downregulated genes, whereas orange dots represent significantly upregulated genes. (b) Heatmap diagram for the Top 5 upregulated and Top 5 downregulated DEGs in the disease group. (c) Venn diagram displaying the overlap of DEGs and RHO GTPase‐related genes (RGPRGs). (d) The Top 5 significantly enriched Gene Ontology (GO) terms in biological process (BP), cellular component (CC), and molecular function (MF) terms for candidate genes. (e) The Top 10 significantly enriched Kyoto Encyclopedia of Genes and Genomes (KEGG) pathways for candidate genes. (f) Protein–protein interaction (PPI) network of candidate genes.

Next, GO enrichment analysis of the candidate genes yielded 514 significantly enriched terms: 419 biological process (BP), 38 cellular component (CC), and 57 molecular function (MF) terms (Table S4). At the BP level, enrichment analysis indicated that the candidate genes were chiefly involved in the dynamic regulation of cellular structures, namely in cell shape regulation and actin filament‐related processes. At the CC level, structures associated with candidate genes (e.g., phagocytic vesicles) were identified as critical carriers for cellular material transport and functional execution. At the MF level, functional enrichment revealed the candidate genes were primarily enriched in signaling and enzymatic activities, notably NADPH oxidase activity and GTPase regulation (Figure 1d). A total of 19 significantly enriched KEGG pathways were identified (Table S5). Significant pathway enrichments for the candidate genes included the chemokine signaling pathway and leukocyte transendothelial migration; the synergistic action of these pathways was found to participate in the regulation of immune function and cellular processes, thereby driving the pathological progression of OA (Figure 1e).

The PPI analysis included all 30 candidate genes and yielded 146 protein‐level interactions (Figure 1f). This network indicated extensive connectivity among the candidate genes in relation to OA.

### 3.2. Machine Learning‐Based Screening of Biomarkers for Clinical Diagnosis of OA

The 30 candidate genes were filtered with two machine learning approaches. Boruta retained 24 Boruta_candidate biomarkers (Figure 2a). The optimized XGBoost model retained 16 XGBoost_candidate biomarkers (Figure 2b). Intersecting the two outputs produced 14 candidate biomarkers (Figure 2c) (Table S6). ROC evaluation of these 14 genes identified VAV1, STMN2, and RND1 as the three key biomarkers (Figure 2d). Diagnostic performance was maintained across the two datasets, as illustrated by VAV1 (AUC 0.960 in the training set and 0.789 in the validation set). Expression validation further showed higher VAV1 and STMN2 levels in OA samples (*p* < 0.05), whereas RND1 expression was higher in controls (*p* < 0.001; Figure 2e,f). Accordingly, VAV1, STMN2, and RND1 were retained as the final biomarkers.

**Figure 2:**
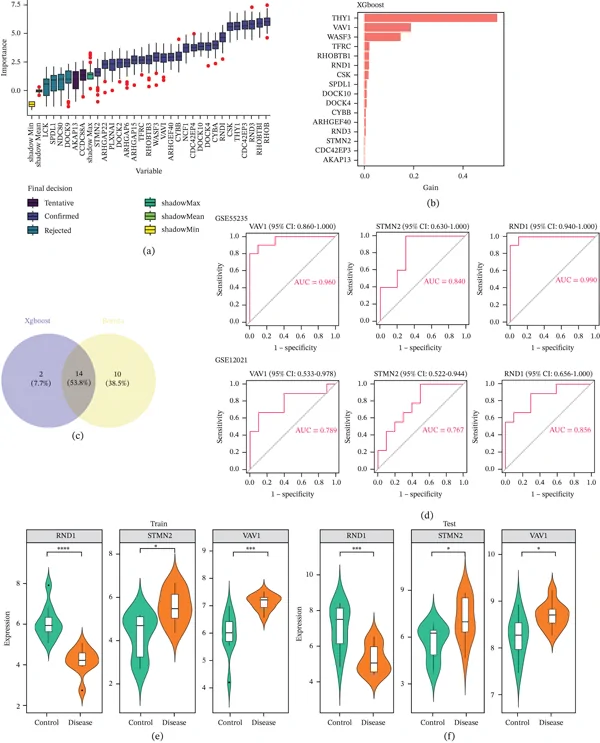
Identification of biomarkers. (a) The 24 Boruta_candidate biomarkers were determined by the Boruta algorithm. (b) The 16 XGBoost_candidate biomarkers were determined by the Extreme Gradient Boosting (XGBoost) algorithm. (c) Venn diagram displaying the overlap of Boruta_candidate biomarkers and XGBoost_candidate biomarkers. (d) The receiver operating characteristic (ROC) curves of VAV1, STMN2, and RND1 in both the training set and validation set. (e) Expression levels of VAV1, STMN2, and RND1 in the training set. (f) Expression levels of VAV1, STMN2, and RND1 in the validation set. ∗*p* < 0.05, ∗∗∗*p* < 0.001, ∗∗∗∗*p* < 0.0001.

### 3.3. The Construction of the ANN Model and the Evaluation of the ROC Curve

An ANN was constructed to test whether the three biomarkers could separate OA samples from controls (Figure 3a). ROC analysis showed AUCs of 0.940 in the training cohort and 0.833 in the validation cohort, indicating good discriminatory performance (Figure 3b).

**Figure 3:**
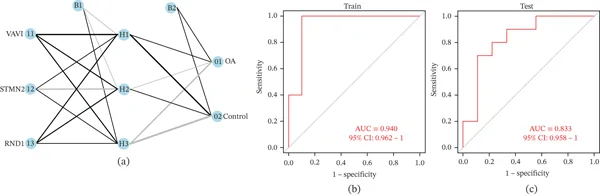
The artificial neural network (ANN) model and the evaluation of the ROC curve. (a) The visualization of the ANN model. (b) The evaluation results of the ROC curve in the training set. (c) The evaluation results of the ROC curve in the validation set.

### 3.4. GSEA of Biomarkers

GSEA was used to characterize pathways linked to VAV1, STMN2, and RND1. After Benjamini–Hochberg correction, 34 pathways for VAV1, 7 for STMN2, and 31 for RND1 met the significance thresholds of BH‐adjusted *p* < 0.05 and | | > 1 (Figure 4a–c; Tables S7, S8, and S9). Five KEGG pathways—lysosome, spliceosome, cell adhesion molecules, Fc gamma R‐mediated phagocytosis, and adipocytokine signaling—were significant for all three genes (Table S10). Together, these enrichment patterns connect the biomarkers with OA‐related processes involving immune regulation, cell adhesion, lysosomal function, RNA processing, and metabolic signaling.

**Figure 4:**
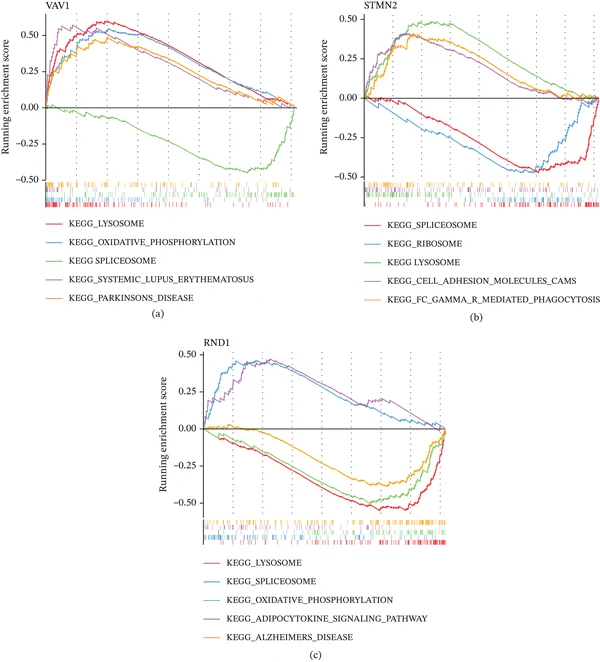
Gene set enrichment analysis (GSEA) of biomarkers. (a) GSEA of VAV1. (b) GSEA of STMN2. (c) GSEA of RND1. Genes were ranked in descending order according to their Spearman correlation coefficients with the expression of each biomarker. The upper panel displays the running enrichment score profiles of representative KEGG pathways. A positive NES means pathway genes are enriched among genes positively correlated with biomarker expression; negative NES means the opposite. Middle‐panel vertical ticks indicate where pathway genes fall in the ranked list, and the lower panel shows how the ranking metric is distributed.

### 3.5. Immune Cell Infiltration

Immune infiltration analysis was performed to investigate the immune cell profiles in the training set. A heatmap was used to visualize abundance scores of 28 immune cell types (Figure 5a). Via the Wilcoxon rank‐sum test, 14 immune cell types showed significant abundance differences (*p* < 0.05) between groups (Figure 5b). The observed immune heterogeneity suggests a key role in OA progression. Notably, VAV1, STMN2, and RND1 were strongly correlated with myeloid‐derived suppressor cells (MDSCs), natural killer (NK) cells, and Type 2 helper cells, respectively (all *p* < 0.01), indicating these biomarkers may drive OA pathology by modulating specific DIICs and contributing to immune microenvironment imbalance (Figure 5c, Tables S11 and S12).

**Figure 5:**
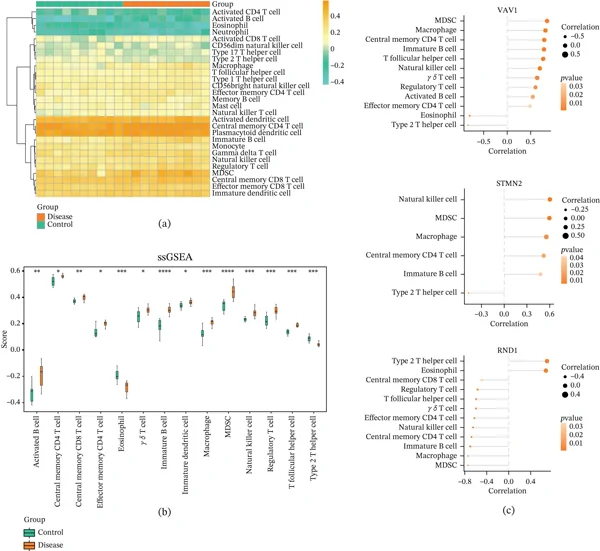
Immune infiltration analysis. (a) Heatmap of abundance scores of 28 immune cell types in the disease group and control group. (b) Boxplot of differences in abundance scores of 14 types of differentially infiltrated immune cells (DIICs) between the disease group and the control group, ∗*p* < 0.05, ∗∗*p* < 0.01, ∗∗∗*p* < 0.001, ∗∗∗∗*p* < 0.0001. (c) Lollipop plot of correlations between biomarkers and DIICs.

### 3.6. Construction of Molecular Regulatory Networks

Networks of mRNA–miRNA and mRNA‐TF interactions were constructed to elucidate the molecular regulation of the biomarkers (Figure 6a,b). A total of 5, 156, and 57 miRNAs with potential interactions with VAV1, STMN2, and RND1 were predicted, respectively (Table S13). Similarly, 10, 7, and 12 TFs with potential regulatory effects on VAV1, STMN2, and RND1 were identified, respectively (Table S14). It was noteworthy that the transcription factor FOXC1 was predicted to exert potential regulatory effects on all three biomarkers. The aforementioned results suggested that these biomarkers may influence the pathological progression of OA by participating in its molecular regulatory mechanisms.

**Figure 6:**
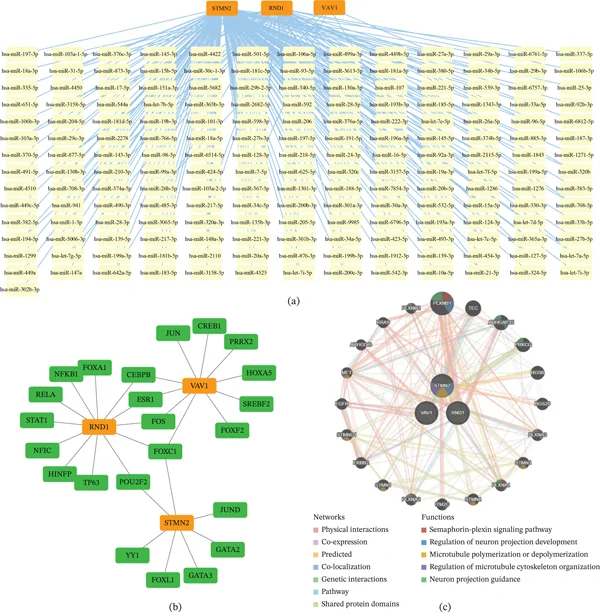
Interaction network of biomarkers. (a) Messenger RNA (mRNA)‐MicroRNA (miRNA) network. (b) mRNA‐transcription factors (TFs) network. (c) Gene Multiple Association Network Integration Algorithm (GeneMANIA) network.

A GeneMANIA network was additionally generated to examine functional relationships between the biomarkers and related genes (Figure 6c). The resulting network was dominated by physical interactions. STMN2‐associated connections were particularly enriched for regulation of neuron projection development, microtubule polymerization or depolymerization, and regulation of microtubule cytoskeleton organization. These network‐level associations provide functional context for the potential involvement of the identified biomarkers in OA.

### 3.7. Potential Drugs Targeting the Biomarkers

Drug prediction analysis was carried out in search of potential therapeutics targeting the biomarkers. A total of 4 potential drugs targeting VAV1, 11 targeting RND1, and 4 targeting STMN2 were predicted. Notably, anisomycin was found to simultaneously target both VAV1 and RND1 (Figure 7a). Favorable affinity was found between VAV1 and 6‐mercaptopurine (−9.3 kcal/mol), STMN2 and tolnaftate (−7.0 kcal/mol), as well as RND1 and primaquine (−6.9 kcal/mol) (Figure 7b).

**Figure 7:**
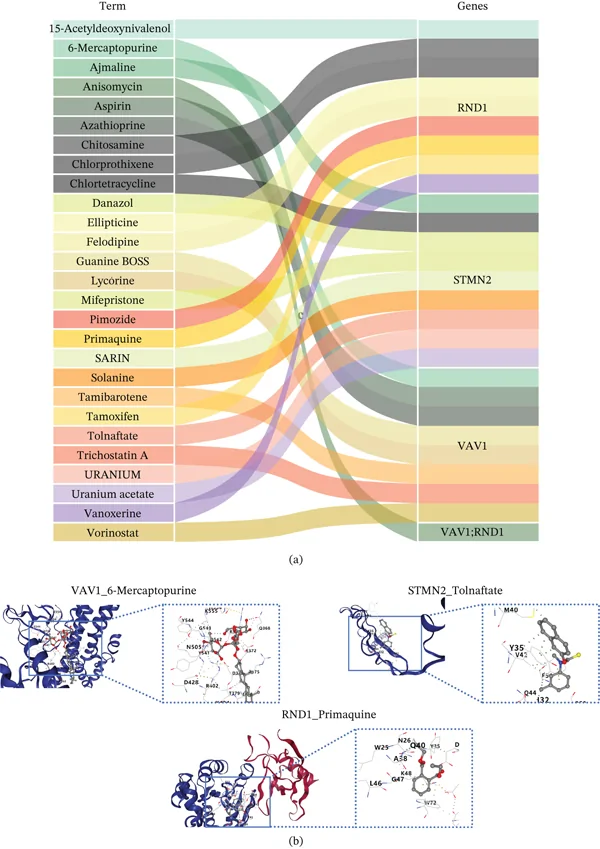
Drug prediction and molecular docking. (a) Sankey diagram between potential drugs and their targeted biomarkers. (b) Molecular docking analysis of drugs and biomarkers (VAV1, STMN2, and RND1). The three‐dimensional protein structure of the key target appears on the left, whereas the right shows the active ingredient′s 3D structure for the targeted drug; between them, a red‐and‐gray ball‐and‐stick model depicts the drug′s molecular architecture. The red dotted line indicates hydrogen bonds, and the blue dotted line indicates hydrophobic interactions. The specific amino acids of the key target are shown around. The blue thick line box represents the magnified docking site, and the letters and numbers in it represent the amino acid residues at specific positions.

### 3.8. Identification of Key Cells in OA

After quality control, the single‐cell dataset was reduced from 10,657 cells and 20,490 genes to 10,293 cells while retaining 20,490 genes (Figure S1). The 2000 most variable genes were identified, with the Top 10 displayed in Figure S2. Following normalization, the Top 30 PCs were selected for downstream analyses (Figures S3 and S4). UMAP based on these 30 PCs separated the cells into 12 clusters (Figure 8a). Cell types were assigned using the following marker sets: fibroblasts (LUM, DCN, FGF7), macrophages (CD68, MARCO, MSR1, CD163), monocytes (CD14, LYZ, S100A9), endothelial cells (CDH5, KLF9, VWF, PECAM1), smooth muscle cells (SMCs; RGS5, ACTA2, TAGLN), T cells (CD2, CD3D, CD3E), mast cells (TPSAB1, CAP3, RGS13), and B cells (CD79B, MS4A1, CD79A) (Figure 8b). Distinct marker‐expression patterns supported these annotations (Figure 8c). Fibroblasts represented a significantly larger fraction of OA cells than the other annotated cell types (Figure 8d), and the biomarkers were expressed in fibroblasts (Figure 8e). On this basis, fibroblasts were selected as the key cell population.

**Figure 8:**
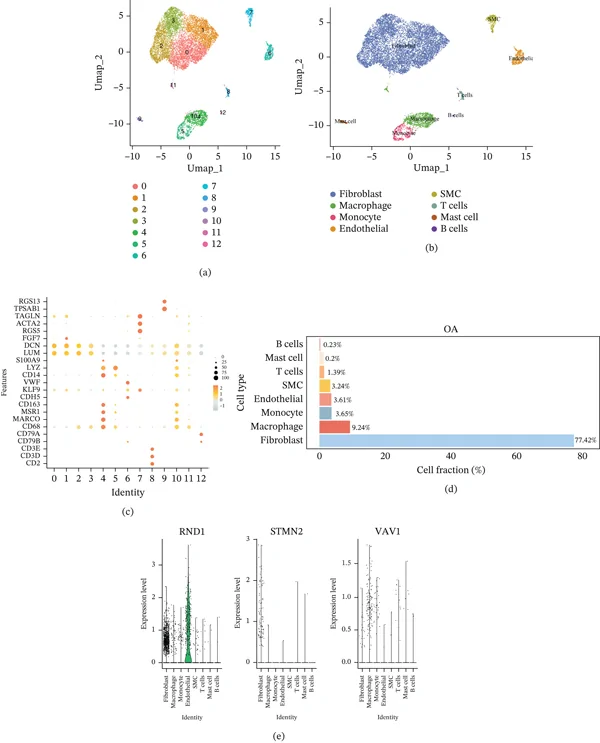
Single‐cell analysis of biomarker expression profiles. (a) UMAP‐based dimensionality reduction and clustering yielded a classification map of cell clusters, color‐coded by cluster identity. (b) UMAP plot showing the annotation of cell clusters into different cell types based on marker genes. (c) Dot plot of cell annotation based on different marker genes. (d) The proportions of different cell types. (e) The expression levels of biomarkers across the eight cell types.

### 3.9. Cell–Cell Interaction Analysis

Cell–cell communication analysis was used to compare the number and strength of interactions across the eight annotated cell types. Fibroblast‐endothelial communication showed a relatively high number and strength of interactions (Figure 9a), indicating particularly strong intercellular signaling between these populations in the OA microenvironment. Communication between fibroblasts and the remaining cell types was mainly associated with the MIF‐(CD74 + CD44) pathway (Figure 9b).

**Figure 9:**
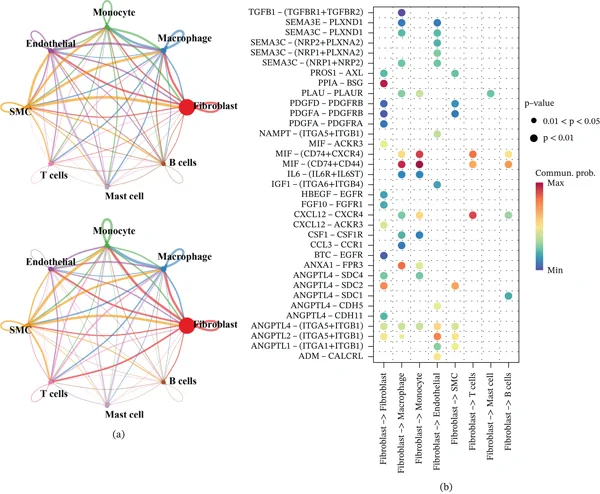
Cell–cell communication analysis. (a) The number and strength of interactions in cellular communication networks. (b) The receptor‐ligand communication weights in fibroblasts.

### 3.10. Pseudotime and Trajectory Analysis

Pseudotime analysis was then applied to reconstruct fibroblast differentiation. Eight fibroblast subpopulations were resolved (Figure 10a). The dark‐to‐light blue pseudotime gradient represented progression from earlier to later states, with the darkest cells located at the beginning of the trajectory and the lightest blue cells on the right at the latest stage (Figure 10b). The trajectory contained nine differentiation states; the purple state represented the earliest state (Figure 10c). Subpopulations 1 and 3 were positioned at terminal differentiation stages (Figure 10d).

**Figure 10:**
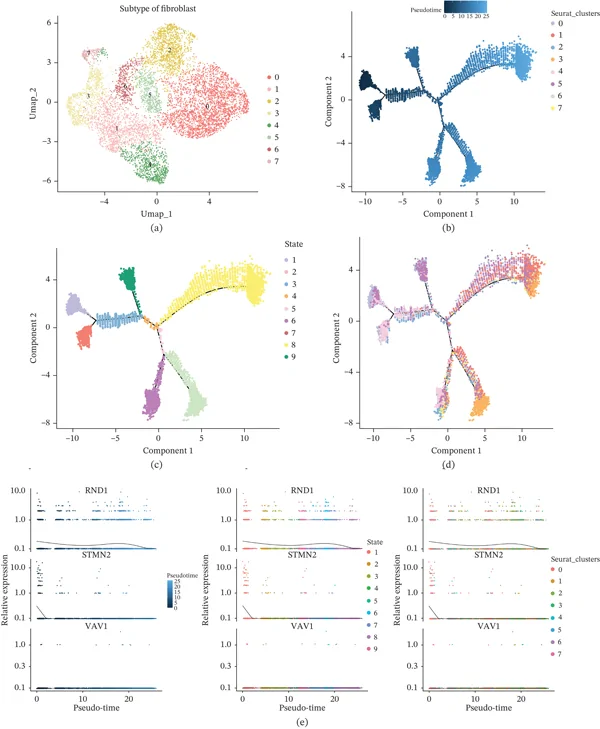
Pseudotime analysis. (a) UMAP diagram of fibroblast clustering analysis. (b) Pseudotemporal analysis of the temporal differences in cell differentiation. (c) Pseudotemporal analysis of the differentiation states of cells at various stages of key cells. (d) Pseudotemporal analysis of the cell differentiation in different subclusters. (e) Changes in the expression levels of biomarkers during fibroblast differentiation.

Expression analysis revealed stage‐specific roles: RND1 decreased early then fluctuated (mid‐late), STMN2 declined sharply early, and VAV1 remained stable, suggesting distinct regulatory functions during fibroblast differentiation (Figure 10e).

### 3.11. Pathway Activity and Metabolic Pathway Characteristics

To dissect the functional landscape of fibroblast subpopulations in OA samples, ReactomeGSA‐based pathway activity analysis was performed. A total of 1740 Reactome pathways were evaluated across the fibroblast subpopulations (Table S15). These pathways were ranked according to the range of their mean activity scores across subpopulations, calculated as the difference between the maximum and minimum scores. To reduce redundancy caused by shared gene members, pathways with a Jaccard similarity ≥ 0.30 were clustered (Figure S5a), and a total of 687 pathways with the largest activity‐score range in each cluster were retained as the representative pathway (Table S16, Figure S5b). After the pathway‐collapsing analysis, the Top 10 pathways showing the largest variation in activity scores are presented in Figure 11a. These pathways broadly encompassed cell–cell interactions, inflammatory and innate immune signaling, transcriptional regulation, and metabolic processes.

**Figure 11:**
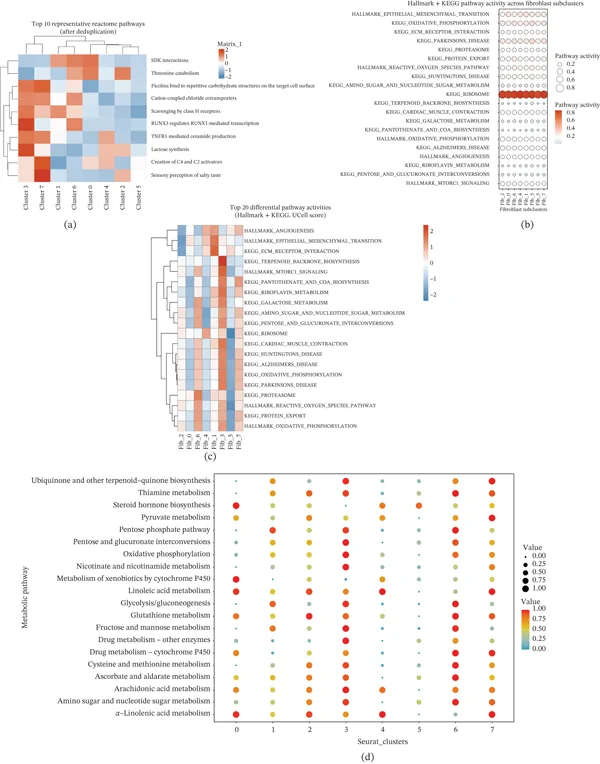
Functional and metabolic pathway enrichment analysis. (a) Colors represent the enrichment levels of different pathways. The closer the color is to orange, the higher the enrichment level of the pathway in the cell population; the closer the color is to green, the lower the enrichment level. (b) Bubble plot showing the activity of the Top 20 Hallmark and KEGG pathways across fibroblast subpopulations. (c) Heatmap of the Top 20 differential Hallmark and KEGG pathways across fibroblast subpopulations based on UCell scores. (d) Metabolism of eight subpopulations of fibroblasts.

To further evaluate pathway activity using alternative gene set collections, Hallmark and KEGG pathways were analyzed using UCell. Differential analysis revealed significant variation in pathway activity among fibroblast subpopulations after Benjamini–Hochberg correction (*p* adj < 0.05). The Top 20 pathways ranked by the range of their mean activity scores included 5 Hallmark and 15 KEGG pathways and were presented in Figure 11b,c and Table S17. These pathways were mainly associated with epithelial–mesenchymal transition, extracellular matrix interactions, oxidative phosphorylation and reactive oxygen species (ROS) responses, protein synthesis and degradation, mTORC1 signaling, angiogenesis, and multiple metabolic processes. Collectively, these complementary analyses further demonstrated pronounced functional heterogeneity among fibroblast subpopulations using gene set collections.

Metabolic activity was also compared across fibroblast subsets. The 20 highest‐scoring metabolic pathways are shown in Figure 11d. Clear subtype‐specific differences were observed: Subset 3 showed relatively high activity across most pathways, whereas Subsets 4 and 5 generally exhibited lower activity. Alpha‐linolenic acid metabolism and linoleic acid metabolism were highly enriched in most subsets. These patterns suggest that fatty‐acid metabolic programs may contribute to fibroblast function within the OA microenvironment.

## 4. Discussion

OA is the most common chronic joint disease, characterized by progressive cartilage degeneration and chronic pain, yet its molecular pathogenesis remains incompletely understood [48, 49]. RHO GTPases are well established as key regulators in the initiation and progression of OA. They modulate critical cellular processes, including chondrocyte differentiation, thereby contributing to OA pathogenesis [50, 51]. It has also been reported that another GTPase, Drp1, can influence the progression of OA by regulating mitochondrial fission, mitophagy, ROS accumulation, and NLRP3 inflammasome activation [52]. Utilizing public datasets and bioinformatics analysis, this study identified three RHO GTPase‐related biomarkers with good diagnostic performance for OA—VAV1, STMN2, and RND1. These biomarkers are likely involved in OA pathological processes by regulating key biological functions such as energy metabolism, RNA processing, and immune regulation. Additionally, these biomarkers were closely associated with fibroblasts, implying a potential role in worsening OA joint inflammation and tissue degeneration via RHO GTPase signaling. Overall, our findings deepen insight into OA pathogenesis and may guide future diagnostic and therapeutic development.

In this study, a total of 14 candidate biomarkers were obtained using the Boruta and XGBoost algorithms, and their diagnostic performance was subsequently further evaluated in the training set and an independent validation set. Only VAV1, STMN2, and RND1 achieved AUC > 0.7 in both datasets, indicating relatively stable diagnostic capability and cross‐dataset predictive performance. The remaining 11 candidate biomarkers achieved AUC > 0.7 in only one dataset or failed to reach this threshold in both datasets, suggesting insufficient consistency and generalizability of their diagnostic performance; consequently, they were not designated as the final candidate diagnostic markers. Nevertheless, these candidate biomarkers may still hold potential significance related to OA. For instance, THY1 can mark synovial fibroblast subpopulations with osteogenic and chondrogenic potential, suggesting its possible involvement in synovial repair and cellular heterogeneity [53]. CYBB encodes NOX2, and its aberrant expression may promote cartilage damage through ROS generation and immuno‐inflammation [54]. Additionally, TFRC is involved in iron uptake and ferroptosis, potentially reflecting iron metabolic dysregulation and damage mechanisms in OA chondrocytes [55].

VAV1 is a core member of the Vav family of guanine nucleotide exchange factors (GEFs). Its encoded protein acts as a key activator of RHO GTPases, regulating downstream signaling pathways by catalyzing the exchange of GDP for GTP, thereby influencing biological processes such as cell proliferation, differentiation, migration, cytoskeletal remodeling, and immune responses [56–58]. In OA, the function of VAV1 is cell type‐specific: in chondrocytes, VAV1 is enhanced by EphA4 signaling, activating Rac1/2 and promoting the expression of chondrogenesis‐related genes; in synovial cells, VAV1 is inhibited by EphA4 signaling, thereby indirectly modulating RHO signaling to reduce the secretion of inflammatory factors and MMPs [59]. This study demonstrated that VAV1 was significantly upregulated in OA samples, suggesting its potential as a diagnostic biomarker for the disease. The high expression of VAV1 may reflect an adaptive regulatory response of joint tissues to injury. In articular chondrocytes, VAV1 can be activated by EphA4 signaling, upregulating the Rac1/2 pathway to promote chondrogenesis‐related gene expression—an attempt by the body to initiate self‐repair of damaged cartilage tissue. Simultaneously, as a key node in the RHO GTPase pathway, the upregulation of VAV1 reflects the overall activation state of this pathway in OA. Previous studies have confirmed that members of the RHO GTPase family play central roles in chondrocyte apoptosis, cytoskeletal disorganization, and synovial fibrosis in OA [60, 61]. As VAV1 serves as an upstream activator of this family, its expression level is directly linked to pathway activity.

STMN2, a core member of the Stathmin family, encodes a highly conserved microtubule‐depolymerizing protein involved in cytoskeletal remodeling, cell division, migration, and differentiation by regulating microtubule dynamics [62–65]. In OA, STMN2 exhibits significantly upregulated expression and may be involved in the regulation of synovial inflammation and cartilage degeneration [66, 67]. This study identified the RGPRGs STMN2 as a diagnostic biomarker for OA, showing significantly high expression in OA samples. Given that a core function of RHO GTPases is precisely the regulation of the cytoskeleton and cell migration [68–70], a natural regulatory connection exists between RHO GTPases and STMN2 at the molecular functional level. Therefore, we hypothesize that STMN2‐mediated regulation of microtubule dynamics may directly respond to RHO GTPase pathway activation or interact with RHO GTPases through downstream molecular networks, thereby influencing the pathological behavior of OA‐related cells. The significantly high expression of STMN2 in disease samples may be a key downstream effect following aberrant activation of the RHO GTPase pathway, amplifying microtubule imbalance to promote inflammatory cell infiltration and extracellular matrix degradation, thereby accelerating disease progression.

RND1 is an important member of the RHO GTPase family, encoding a small GTP‐binding protein with unique functionality. Unlike traditional RHO GTPases that require GEFs for activation, RND1 is constitutively active state constitutively without GEF activation. It regulates cytoskeletal rearrangement, adherens junctions, and signal transduction, extensively participating in key biological processes such as cell migration, differentiation, apoptosis, and inflammation [71–74]. In OA, RND1 has been confirmed as a downstream target gene induced by tumor necrosis factor‐alpha (TNF‐*α*), mediating cellular growth, death, and inflammatory stress responses through the NF‐*κ*B and AP‐1 pathways. Its aberrant expression can exacerbate local inflammation and cartilage damage in OA [75]. However, this study found that RND1 can serve as a diagnostic biomarker for OA, with significantly lower expression in OA samples. This may represent an adaptive negative feedback regulation occurring in the late stages of the disease to suppress excessive inflammation. Initial TNF‐*α* stimulation might elevate RND1 to transmit inflammatory signals, but persistent damage could lead to its downregulation in an attempt to mitigate tissue destruction. Paradoxically, the low expression of RND1 may disrupt the normal RHO GTPase‐RND1 regulatory network, leading to inadequate inflammation control and impaired cartilage repair, thereby exacerbating OA.

The NLR signaling pathway is a core component of innate immunity, initiating inflammatory responses through intracellular recognition of damage‐associated molecular patterns (DAMPs) [76, 77]. In OA, its abnormal activation exacerbates joint inflammation and promotes matrix degradation by mediating NLRP3 inflammasome activation [78]. In the NLR signaling pathway, VAV1 and STMN2 were significantly downregulated, whereas RND1 was significantly upregulated. These divergent expression patterns suggest complex negative feedback mechanisms. The downregulation of VAV1 and STMN2 may limit inflammasome assembly by reducing cytoskeletal rearrangement and microtubule remodeling required for NLRP3 activation [79, 80]. Conversely, RND1 upregulation may represent direct negative feedback inhibition, potentially suppressing NLRP3‐related gene transcription through interference with NF‐*κ*B/AP‐1 activity [81]. These coordinated regulatory responses highlight the intricate crosstalk between RHO GTPase signaling and innate immune pathways in OA pathogenesis.

MDSCs constitute a heterogeneous population of immature myeloid cells, characterized by potent immunosuppressive functions mediated through multiple mechanisms, including the secretion of anti‐inflammatory factors and direct inhibition of T‐cell function [82–84]. In OA, MDSCs exhibit functional plasticity: they can exacerbate IL‐17‐mediated inflammation and cartilage degradation by promoting Th17 differentiation, but they can also induce macrophages to polarize toward a reparative M2 phenotype, participating in tissue repair. This dual “proinflammatory versus reparative” characteristic makes them a potential target for OA immunomodulation [85]. This study found that the RGPRG VAV1 showed a significant positive correlation with MDSCs, and MDSC infiltration levels were significantly higher in the disease group. This positive correlation may stem from a regulatory cascade in which VAV1 activation enhances RHO signaling, leading to inflammatory factor release and subsequent MDSC recruitment [86–88]. Notably, both VAV1 and MDSCs possess dual regulatory roles in OA: VAV1 can promote cartilage synthesis via the EphA4/Rac pathway in chondrocytes, whereas MDSCs can also participate in repair by inducing macrophage M2 polarization. Their positive correlation may reflect a joint attempt to counteract disease progression. However, this synergistic effect could also further amplify OA inflammation. The proinflammatory effects of MDSCs are key factors driving cartilage degradation. When high VAV1 expression activates the RHO signaling pathway, this activation increases the secretion of inflammatory factors in synovial cells, potentially forming a vicious cycle: elevated VAV1 expression activates the RHO pathway, leading to inflammatory factor release, which then recruits more MDSCs, subsequently enhancing Th17 cell differentiation and ultimately amplifying inflammation.

Fibroblasts originate from mesenchymal stem cells and serve as the core functional cells constituting connective tissue. They are capable of synthesizing extracellular matrix components such as collagen and elastic fibers [89–91]. During the progression of OA, synovial fibroblast‐like synoviocytes, under inflammatory stimulation, undergo a fibroblast‐to‐myofibroblast transition (FMT), differentiating into myofibroblasts that highly express *α*‐SMA. These cells drive joint fibrosis through excessive collagen secretion, thereby exacerbating joint pathology [92]. This study identifies fibroblasts as key cells in OA, with their proportion significantly increased in lesioned samples. Three markers (VAV1, STMN2, RND1) are abundantly expressed in these cells. VAV1 may participate in FMT by regulating cytoskeletal remodeling and inflammatory signaling [93], whereas STMN2 likely promotes fibroblast migration and inflammatory factor secretion by exacerbating microtubule instability [94]. RND1 exhibits a complex temporal expression pattern: It is upregulated early under TNF‐*α* stimulation but downregulated in the sustained inflammatory phase. This may disrupt the balance of the “RHO GTPase–RND1” signaling pathway, leading to abnormal fibroblast activation. Furthermore, the study observed frequent interactions between fibroblasts and endothelial cells. Activated fibroblasts increase vascular permeability by secreting inflammatory factors [95–97], which in turn accelerates inflammatory cell infiltration and sustains the ongoing FMT process. Pseudotemporal trajectory analysis further reveals the dynamic differentiation process of fibroblasts, which can be categorized into eight subpopulations and nine differentiation states. Among these, subpopulations 1 and 3 represent terminally differentiated groups, likely corresponding to functional myofibroblasts. Previous single‐cell studies have demonstrated that OA synovial fibroblasts exhibit significant functional heterogeneity, with distinct subpopulations characterized by features such as secretion of inflammatory cytokines, extracellular matrix remodeling, fibrosis, and regulation of pain signals. Notably, fibroblast subpopulations from pain‐related sites display stronger inflammatory, fibrotic, and nerve growth‐related transcriptional signatures [98], whereas CD39^+^CD55^−^ fibroblasts exhibit a myofibroblast‐like phenotype and correlate with the severity of knee OA pain [99]. These findings support the notion that the different fibroblast subpopulations and differentiation states observed in our study may represent distinct OA‐related functional phases. Furthermore, previous research has shown that OA synovial fibroblasts can undergo continuous transitions across different functional states, regulated by signals such as TGF‐*β* and WNT [100]. RND1 exhibited a dynamic expression pattern during fibroblast differentiation, characterized by an early decrease, a mid‐phase increase, and a late decrease. RND1 is known to regulate the cytoskeleton, cell adhesion, and cell morphology. Its early decrease may facilitate fibroblast activation and morphological remodeling, whereas the mid‐phase increase may contribute to coordinating cell migration and intercellular communication [101–103]. Given that OA‐related fibroblast‐to‐myofibroblast transformation is typically accompanied by sustained extracellular matrix deposition and tissue remodeling, the late decrease may be associated with the terminal fibrotic phenotype [92]; however, the underlying mechanisms require further validation.

Representative Reactome pathways after de‐redundancy further suggest that OA synovial fibroblast subpopulations may exhibit functional heterogeneity in terms of cell–cell interactions, inflammation and innate immunity, transcriptional regulation, and metabolic status, which is in line with previous single‐cell studies demonstrating that OA synovial fibroblasts can adopt cellular states with distinct inflammatory and tissue‐remodeling functions [104]. Among these pathways, the differential enrichment of the TNFR1‐mediated ceramide production pathway aligns with the notion that TNF signaling may participate in fibroblast inflammatory activation, whereas the pathways of Ficolin recognition and formation of C4 and C2 activators point to the lectin pathway and classical complement response. Indeed, previous studies have detected lectin‐pathway proteins such as M‐ficolin and H‐ficolin in OA synovial fluid, confirmed complement system activation, and demonstrated that OA synovial tissues can express classical complement components including C2 and C4 [105, 106], suggesting that complement‐related immune processes may be involved in the inflammatory microenvironment surrounding distinct fibroblast subpopulations. Additionally, the pathway “RUNX3 regulates RUNX1‐mediated transcription” indicates potential subpopulation differences in transcriptional regulation; RUNX1 has been reported to maintain joint homeostasis and suppress OA progression [107], though its specific role in synovial fibroblasts requires further validation. Metabolic pathways such as threonine catabolism and lactose synthesis may reflect metabolic reprogramming rather than actual lactose synthesis in synovial tissue, as previous work has confirmed glycolytic and mitochondrial abnormalities in OA synovial fibroblasts and shown that metabolic interventions can alter their inflammatory phenotype [108]. Therefore, these findings should be viewed primarily as exploratory clues for interpreting the functional heterogeneity of fibroblasts.

In summary, comprehensive bioinformatics analysis identified VAV1, STMN2, and RND1 as promising biomarkers for early OA diagnosis. These three RHO GTPase‐related biomarkers can accurately predict OA occurrence. Furthermore, all three biomarkers were highly expressed in fibroblasts. Collectively, this study provides a theoretical basis for elucidating the mechanistic roles of RHO GTPase‐related biomarkers in OA pathological processes and provides potential insights for future clinical applications. Nevertheless, several limitations should be acknowledged. Firstly, the sample sizes of GSE55235 and GSE12021 are limited, and both datasets are derived from the same microarray platform, which may restrict the generalizability of the findings across different populations and detection platforms. Secondly, this study is primarily a retrospective bioinformatics analysis based on public databases, and further validation using multicenter, large‐sample, prospective clinical cohorts and independent clinical specimens is currently lacking. Therefore, future research should focus on complementary in vivo and in vitro experiments (e.g., gene knockdown assays) and multicenter validation studies to further substantiate their clinical value.

## Author Contributions

Guoqiang Zou: writing—original draft, writing—review and editing, formal analysis, and methodology. Xufeng Wang: writing—review and editing, software, validation, and investigation. Jing Yu: writing—review and editing, conceptualization, supervision, and project administration.

## Funding

No funding was received for this manuscript.

## Ethics Statement

The authors have nothing to report.

## Consent

The authors have nothing to report.

## Conflicts of Interest

The authors declare no conflicts of interest.

## Supporting Information

Additional supporting information can be found online in the Supporting Information section.

## Supporting information


**Supporting Information 1** Table S1: The 733 RGPRGs collected in this study.

**Supporting Information 2** Table S2: The 801 DEGs identified in the training set.

**Supporting Information 3** Table S3: The 30 candidate genes identified in this study.

**Supporting Information 4** Table S4: The 514 GO terms significantly enriched in candidate genes.

**Supporting Information 5** Table S5: The 19 KEGG pathways significantly enriched in candidate genes.

**Supporting Information 6** Table S6: The 14 candidate biomarkers identified in the training set.

**Supporting Information 7** Table S7: The 52 KEGG pathways significantly enriched with VAV1 identified in this study.

**Supporting Information 8** Table S8: The 37 KEGG pathways significantly enriched with STMN2 identified in this study.

**Supporting Information 9** Table S9: The 47 KEGG pathways significantly enriched with RND1 identified in this study.

**Supporting Information 10** Table S10: The 19 KEGG pathways collectively enriched by biomarkers identified in this study.

**Supporting Information 11** Table S11: Correlation coefficients between biomarkers and DIICs.

**Supporting Information 12** Table S12: *p* values between biomarkers and DIICs.

**Supporting Information 13** Table S13: miRNAs with potential interactions with biomarkers.

**Supporting Information 14** Table S14: TFs with potential regulatory effects on biomarkers.

**Supporting Information 15** Table S15: The 1740 pathways with differential enrichment among different fibroblast subpopulations identified in this study.

**Supporting Information 16** Table S16: Representative Reactome pathways after pathway collapsing.

**Supporting Information 17** Table S17: Differential hallmark and KEGG pathway activity across fibroblast subpopulations based on UCell analysis.

**Supporting Information 18** Figure S1: Violin plots of nCount RNA, nFeature RNA, and percent.mt in single‐cell data before and after quality control.

**Supporting Information 19** Figure S2: Scatter plot showing mean gene expression and normalized variance in a single‐cell dataset.

**Supporting Information 20** Figure S3: Principal component analysis (PCA) plot.

**Supporting Information 21** Figure S4: Elbow plot of variance explained by principal components from PCA.

**Supporting Information 22** Figure S5: Reactome pathway redundancy analysis. (a) Jaccard similarity matrix of the Top 20 Reactome pathways based on shared gene members. (b) Representative Reactome pathways after pathway collapsing, ranked by the maximum–minimum difference in pathway activity across fibroblast subpopulations; color indicates the number of merged pathways.

## Data Availability

The datasets supporting the conclusions of this article are available in the [GEO] repository, [https://www.ncbi.nlm.nih.gov/geo/].

## References

[1] Yin B. , Ni J. , Witherel C. E. , Yang M. , Burdick J. A. , Wen C. , and Wong S. H. D. , Harnessing Tissue-Derived Extracellular Vesicles for Osteoarthritis Theranostics, Theranostics. (2022) 12, no. 1, 207–231, 10.7150/thno.62708, 34987642. PMC8690930 34987642

[2] Cho Y. , Jeong S. , Kim H. , Kang D. , Lee J. , Kang S.-B. , and Kim J. H. , Disease-Modifying Therapeutic Strategies in Osteoarthritis: Current Status and Future Directions, Experimental & Molecular Medicine. (2021) 53, no. 11, 1689–1696, 10.1038/s12276-021-00710-y, 34848838. PMC8640059 34848838

[3] Nielsen R. L. , Monfeuga T. , Kitchen R. R. , Egerod L. , Leal L. G. , Schreyer A. T. H. , Gade F. S. , Sun C. , Helenius M. , Simonsen L. , Willert M. , Tahrani A. A. , McVey Z. , and Gupta R. , Data-Driven Identification of Predictive Risk Biomarkers for Subgroups of Osteoarthritis Using Interpretable Machine Learning, Nature Communications. (2024) 15, no. 1, 10.1038/s41467-024-46663-4, 38561399. PMC10985086 38561399

[4] Warmink K. , Vinod P. , Korthagen N. M. , Weinans H. , and Rios J. L. , Macrophage-Driven Inflammation in Metabolic Osteoarthritis: Implications for Biomarker and Therapy Development, International Journal of Molecular Sciences. (2023) 24, no. 7, 10.3390/ijms24076112, 37047082. PMC10094694 37047082

[5] Hu X. , Ni S. , Zhao K. , Qian J. , and Duan Y. , Bioinformatics-Led Discovery of Osteoarthritis Biomarkers and Inflammatory Infiltrates, Frontiers in Immunology. (2022) 13, 871008, 10.3389/fimmu.2022.871008, 35734177. PMC9207185 35734177

[6] Dantas L. O. , de Fátima Salvini T. , and McAlindon T. E. , Knee Osteoarthritis: Key Treatments and Implications for Physical therapy, Brazilian Journal of Physical Therapy. (2021) 25, no. 2, 135–146, 10.1016/j.bjpt.2020.08.004, 33262080. PMC7990728 33262080

[7] Zhang H. , Jiang X. , Bai L. , Chen J. , Luo W. , Ma J. , and Ma X. , Vitamin C Intake and Osteoarthritis: Findings of NHANES 2003-2018 and Mendelian Randomization Study, Frontiers in Nutrition. (2024) 11, 1409578, 10.3389/fnut.2024.1409578, 39507896. PMC11537885 39507896

[8] Bement W. M. , Goryachev A. B. , Miller A. L. , and von Dassow G. , Patterning of the Cell Cortex by rho GTPases, Nature Reviews Molecular Cell Biology. (2024) 25, no. 4, 290–308, 10.1038/s41580-023-00682-z, 38172611. PMC12706751 38172611

[9] Crosas-Molist E. , Samain R. , Kohlhammer L. , Orgaz J. L. , George S. L. , Maiques O. , Barcelo J. , and Sanz-Moreno V. , Rho GTPase Signaling in Cancer Progression and Dissemination, Physiological Reviews. (2022) 102, no. 1, 455–510, 10.1152/physrev.00045.2020, 34541899. 34541899

[10] Panchal K. and Tiwari A. K. , Miro (Mitochondrial rho GTPase), a Key Player of Mitochondrial Axonal Transport and Mitochondrial Dynamics in Neurodegenerative Diseases, Mitochondrion. (2021) 56, 118–135, 10.1016/j.mito.2020.10.005, 33127590. 33127590

[11] Tanaka R. and Yamada K. , Genomic and Reverse Translational Analysis Discloses a Role for Small GTPase Rho A Signaling in the Pathogenesis of Schizophrenia: Rho-Kinase as a Novel Drug Target, International Journal of Molecular Sciences. (2023) 24, no. 21, 15623, 10.3390/ijms242115623, 37958606. PMC10648424 37958606

[12] Malmhäll-Bah E. , Andersson K. M. E. , Erlandsson M. C. , Akula M. K. , Brisslert M. , Wiel C. , el Zowalaty A. E. , Sayin V. I. , Bergö M. O. , and Bokarewa M. I. , Rho-GTPase Dependent Leukocyte Interaction Generates Pro-Inflammatory Thymic Tregs and Causes Arthritis, Journal of Autoimmunity. (2022) 130, 102843, 10.1016/j.jaut.2022.102843, 35643017. 35643017

[13] Dipankar P. , Kumar P. , Dash S. P. , and Sarangi P. P. , Functional and Therapeutic Relevance of Rho GTPases in Innate Immune Cell Migration and Function During Inflammation: An In Silico Perspective, Mediators of Inflammation. (2021) 2021, 6655412, 10.1155/2021/6655412, 33628114. PMC7896857 33628114

[14] Dong J. , Zhang K.-J. , Li G.-C. , Chen X.-R. , Lin J.-J. , Li J.-W. , Lv Z. Y. , Deng Z. Z. , Dai J. , Cao W. , and Jiang Q. , CDDO-im Ameliorates Osteoarthritis and Inhibits Chondrocyte Apoptosis in Mice via Enhancing Nrf2-Dependent Autophagy, Acta Pharmacologica Sinica. (2022) 43, no. 7, 1793–1802, 10.1038/s41401-021-00782-6, 34754093. PMC9253092 34754093

[15] Horváth E. , Sólyom Á. , Székely J. , Nagy E. E. , and Popoviciu H. , Inflammatory and Metabolic Signaling Interfaces of the Hypertrophic and Senescent Chondrocyte Phenotypes Associated With Osteoarthritis, International Journal of Molecular Sciences. (2023) 24, no. 22, 16468, 10.3390/ijms242216468, 38003658. PMC10671750 38003658

[16] Luo M. , Du X.-M. , and Zhou X.-D. , Developments in Research on the Relationship Between Matrix Metalloproteinases and Osteoarthritis, Journal of Sichuan University (Medical Sciences). (2023) 54, no. 1, 77–82, 10.12182/20230160110, 36647647. PMC10409029 36647647

[17] Nieuwstraten J. , Riester R. , Hofmann U. K. , Guilak F. , and Danalache M. , Matrix Metalloproteinases Accelerate Pericellular Matrix Breakdown and Disrupt Mechanotransduction in Osteoarthritis, Acta Biomaterialia. (2025) 195, 73–82, 10.1016/j.actbio.2025.02.034. 39956307

[18] Chen Q. , Yao Y. , Chen K. , Chen X. , Li B. , Li R. , Mo L. , Hu W. , Zhang M. , Wang Z. , Wu Y. , Wu Y. , and Liu F. , Aberrant Activation of TGF-*β*1 Induces High Bone Turnover via Rho GTPases-Mediated Cytoskeletal Remodeling in Camurati-Engelmann Disease, Frontiers in Endocrinology. (2022) 13, 913979, 10.3389/fendo.2022.913979, 36325441. PMC9621586 36325441

[19] Yang J. , Wang L. , Zhang Z. , Sun Q. , and Zhang Y. , Downregulation of HAS-2 Regulates the Chondrocyte Cytoskeleton and Induces Cartilage Degeneration by Activating the RhoA/ROCK Signaling Pathway, International Journal of Molecular Medicine. (2023) 52, no. 1, 10.3892/ijmm.2023.5260, 37232339. PMC10241152 37232339

[20] Shi H. , Li S. , Geng Y. , Fan H. , Zhang R. , Zhang Y. , Pan J. , Song G. , Ge L. , Xie T. , and Wang L. , Euphorbia Factor L3 Ameliorates Rheumatoid Arthritis by Suppressing the Inflammatory Response by Targeting Rac Family Small GTPase 1, Bioengineered. (2022) 13, no. 4, 10984–10997, 10.1080/21655979.2022.2066761, 35475473. PMC9208460 35475473

[21] Hu Z. , Chen Y. , Zhu S. , Feng X. , Zhang B. , and Huang J. , Sonic Hedgehog Promotes Proliferation and Migration of Fibroblast-Like Synoviocytes in Rheumatoid Arthritis via Rho/ROCK Signaling, Journal of Immunology Research. (2022) 2022, 3423692, 10.1155/2022/3423692, 35785032. PMC9242744 35785032

[22] Qu Y. , Wang Y. , Wang S. , Yu X. , He Y. , Lu R. , Chen S. , Meng C. , Xu H. , Pei W. , Ni B. , Zhang R. , Huang X. , and You H. , A Comprehensive Analysis of Single-Cell RNA Transcriptome Reveals Unique SPP1+ Chondrocytes in Human Osteoarthritis, Computers in Biology and Medicine. (2023) 160, 106926, 10.1016/j.compbiomed.2023.106926, 37141654. 37141654

[23] Hao T. , Pei Z. , Hu S. , Zhao Z. , He W. , Wang J. , Jiang L. , Ariben , Wu L. , Yang X. , Wang L. , Wu Y. , Chen X. , Li Q. , Yang H. , Li S. , Wang X. , Sun M. , and Zhang B. , Identification of Osteoarthritis-Associated Chondrocyte Subpopulations and Key Gene-Regulating Drugs Based on Multi-Omics Analysis, Scientific Reports. (2025) 15, no. 1, 10.1038/s41598-025-90694-w, 40216809. PMC11992032 40216809

[24] Zhang B. , Xu L. , Ji Y. , Li J. , Liu L. , Li L. , Zhuo Z. , and Zheng Z. , RHOGTPase-Related Gene Signature Predicts Prognosis, Immunotherapy Response, and Chemotherapy Sensitivity in Colon Cancer, Applied Biochemistry and Biotechnology. (2025) 197, no. 7, 4702–4718, 10.1007/s12010-025-05262-9, 40347353. 40347353

[25] Wang Y. , Bin T. , Tang J. , Xu X.-J. , Lin C. , Lu B. , and Sun T. T. , Construction of an Acute Myeloid Leukemia Prognostic Model Based on m6A-Related Efferocytosis-Related Genes, Frontiers in Immunology. (2023) 14, 1268090, 10.3389/fimmu.2023.1268090, 38077322. PMC10704160 38077322

[26] Yang D. , He Y. , Wu B. , Liu R. , Wang N. , Wang T. , Luo Y. , Li Y. , and Liu Y. , Predictions of the Dysregulated Competing Endogenous RNA Signature Involved in the Progression of Human Lung Adenocarcinoma, Cancer Biomarkers. (2020) 29, no. 3, 399–416, 10.3233/CBM-200133, 32741804. PMC12662519 32741804

[27] Zhou W. , Li H. , Zhang J. , Liu C. , Liu D. , Chen X. , Ouyang J. , Zeng T. , Peng S. , Ouyang F. , Long Y. , and Li Y. , Identification and Mechanism Analysis of Biomarkers Related to Butyrate Metabolism in COVID-19 Patients, Annals of Medicine. (2025) 57, no. 1, 2477301, 10.1080/07853890.2025.2477301, 40074706. PMC11905318 40074706

[28] Song H. , Zhou H. , Yang S. , and He C. , Combining Mendelian Randomization Analysis and Network Toxicology Strategy to Identify Causality and Underlying Mechanisms of Environmental Pollutants With Glioblastoma: A Study of Methyl-4-Hydroxybenzoate, Ecotoxicology and Environmental Safety. (2024) 287, 117311, 10.1016/j.ecoenv.2024.117311, 39536568. 39536568

[29] Twait E. L. , Andaur Navarro C. L. , Gudnason V. , Hu Y.-H. , Launer L. J. , and Geerlings M. I. , Dementia Prediction in the General Population Using Clinically Accessible Variables: A Proof-Of-Concept Study Using Machine Learning. The AGES-Reykjavik Study, BMC Medical Informatics and Decision Making. (2023) 23, no. 1, 10.1186/s12911-023-02244-x, 37641038. PMC10463542 37641038

[30] Peng H. , Lv B. , Du J. , Huang Y. , Cui Q. , Cui C. , and Jin H. , Identification of Metabolism-Related Hub Genes in Heart Failure via Comprehensive Transcriptome Analysis, Genes.(2025) 16, no. 3, 10.3390/genes16030305, 40149456. PMC11941980 40149456

[31] Zhong X. , Shang J. , Zhang R. , Zhang X. , Yu L. , Niu H. , and Duan X. , Explore the Shared Molecular Mechanism Between Dermatomyositis and Nasopharyngeal Cancer by Bioinformatic Analysis, PLoS One. (2024) 19, no. 5, e0296034, 10.1371/journal.pone.0296034, 38753689. PMC11098312 38753689

[32] Li Z. , Guo H. , Ge Y. , Li X. , Dong F. , and Zhang F. , Decoding Alzheimer′s Disease With Depression: Molecular Insights and Therapeutic Target, Journal of Cellular and Molecular Medicine. (2025) 29, no. 5, e70454, 10.1111/jcmm.70454, 40074694. PMC11903198 40074694

[33] Qing M. , Yang D. , Shang Q. , Li W. , Zhou Y. , Xu H. , and Chen Q. , Humoral Immune Disorders Affect Clinical Outcomes of Oral Lichen Planus, Oral Diseases. (2024) 30, no. 4, 2337–2346, 10.1111/odi.14667, 37392455. 37392455

[34] Zhang J. , Wen Z. Y. , Li Y. X. , Sun H. P. , and Zheng Y. Y. , Identification and Validation of Key Genes Related to Apoptosis in Multiple Organ Dysfunction Syndrome, European Journal of Medical Research. (2025) 30, no. 1, 10.1186/s40001-025-03292-x, 41204291. PMC12595649 41204291

[35] Zhou J. , Huang J. , Li Z. , Song Q. , Yang Z. , Wang L. , and Meng Q. Q. , Identification of Aging-Related Biomarkers and Immune Infiltration Characteristics in Osteoarthritis Based on Bioinformatics Analysis and Machine Learning, Frontiers in Immunology. (2023) 14, 1168780, 10.3389/fimmu.2023.1168780, 37503333. PMC10368975 37503333

[36] Zhang Y. , Miao Y. , Tan J. , Chen F. , Lei P. , and Zhang Q. , Identification of Mitochondrial Related Signature Associated With Immune Microenvironment in Alzheimer′s Disease, Journal of Translational Medicine. (2023) 21, no. 1, 10.1186/s12967-023-04254-9, 37434203. PMC10334674 37434203

[37] Xie Q. , Zhang X. , Liu F. , Luo J. , Liu C. , Zhang Z. , Yang Y. , and Li X. , Identification and Verification of Immune-Related Genes for Diagnosing the Progression of Atherosclerosis and Metabolic Syndrome, BMC Cardiovascular Disorders. (2024) 24, no. 1, 10.1186/s12872-024-04026-3, 39095691. PMC11295872 39095691

[38] Tan Z. , Chen X. , Zuo J. , Fu S. , Wang H. , and Wang J. , Comprehensive Analysis of scRNA-seq and Bulk RNA-seq Reveals Dynamic Changes in the Tumor Immune Microenvironment of Bladder Cancer and Establishes a Prognostic Model, Journal of Translational Medicine. (2023) 21, no. 1, 10.1186/s12967-023-04056-z, 36973787. PMC10044739 36973787

[39] Cao G. , Xuan X. , Li Y. , Hu J. , Zhang R. , Jin H. , and Dong H. , Single-Cell RNA Sequencing Reveals the Vascular Smooth Muscle Cell Phenotypic Landscape in Aortic Aneurysm, Cell Communication and Signaling. (2023) 21, no. 1, 10.1186/s12964-023-01120-5, 37189183. PMC10184349 37189183

[40] Hu X. , Li Z. , Ji M. , Lin Y. , Chen Y. , and Lu J. , Identification of Cellular Heterogeneity and Immunogenicity of Chondrocytes via Single-Cell RNA Sequencing Technique in Human Osteoarthritis, Frontiers in Pharmacology. (2022) 13, 1004766, 10.3389/fphar.2022.1004766, 36249797. PMC9562112 36249797

[41] Fang Z. , Li J. , Cao F. , and Li F. , Integration of scRNA-seq and Bulk RNA-Seq Reveals Molecular Characterization of the Immune Microenvironment in Acute Pancreatitis, Biomolecules. (2022) 13, no. 1, 10.3390/biom13010078, 36671463. PMC9855877 36671463

[42] Wang L. , Li X. , Xu C. , Wang D. , Ma C. , Wang Z. , Li Y. , and Li Z. , Unveiling Novel Cell Clusters and Biomarkers in Glioblastoma and Its Peritumoral Microenvironment at the Single-Cell Perspective, Journal of Translational Medicine. (2024) 22, no. 1, 10.1186/s12967-024-05313-5, 38851695. PMC11162569 38851695

[43] Xu K. , Li J. , Qin L. , Liu J. , Yang H. , Dou G. , Ma L. X. , Dong Y. , and Wang Y. , Decoding Ecosystem Heterogeneity and Transcriptional Regulation Characteristics of Multi-Subtype Renal Cell Carcinoma, Heliyon.(2024) 10, no. 13, e33196, 10.1016/j.heliyon.2024.e33196, 39044973. PMC11263639 39044973

[44] Liberzon A. , Subramanian A. , Pinchback R. , Thorvaldsdóttir H. , Tamayo P. , and Mesirov J. P. , Molecular Signatures Database (MSigDB) 3.0, Bioinformatics. (2011) 27, no. 12, 1739–1740, 10.1093/bioinformatics/btr260, 21546393. PMC3106198 21546393

[45] Asakura K. , Occhiuto K. , Tarshis S. , and Dubrowski A. , Designing and Conducting Healthcare Simulations: Contributions From Social Work, Cureus. (2021) 13, no. 7, e16193, 10.7759/cureus.16193, 34367797. PMC8336964 34367797

[46] Ritchie M. E. , Phipson B. , Wu D. , Hu Y. , Law C. W. , Shi W. , and Smyth G. K. , Limma Powers Differential Expression Analyses for RNA-Sequencing and Microarray Studies, Nucleic Acids Research. (2015) 43, no. 7, 10.1093/nar/gkv007, 25605792. PMC4402510 25605792

[47] Qing J. , Li C. , Zhi H. , Zhang L. , Wu J. , and Li Y. , Exploring Macrophage Heterogeneity in IgA Nephropathy: Mechanisms of Renal Impairment and Current Therapeutic Targets, International Immunopharmacology. (2024) 140, 112748, 10.1016/j.intimp.2024.112748, 39106714. 39106714

[48] Hall M. , van der Esch M. , Hinman R. S. , Peat G. , de Zwart A. , Quicke J. G. , Runhaar J. , Knoop J. , van der Leeden M. , de Rooij M. , Meulenbelt I. , Vliet Vlieland T. , Lems W. F. , Holden M. A. , Foster N. E. , and Bennell K. L. , How Does Hip Osteoarthritis Differ From Knee Osteoarthritis?, Osteoarthritis and Cartilage. (2022) 30, no. 1, 32–41, 10.1016/j.joca.2021.09.010. 34600121

[49] Yang L. , Yu X. , Liu M. , and Cao Y. , A Comprehensive Analysis of Biomarkers Associated With Synovitis and Chondrocyte Apoptosis in Osteoarthritis, Frontiers in Immunology. (2023) 14, 1149686, 10.3389/fimmu.2023.1149686, 37545537. PMC10401591 37545537

[50] Fan X. , Ong L. J. , Sun A. R. , and Prasadam I. , From Polarity to Pathology: Decoding the Role of Cell Orientation in Osteoarthritis, Journal of Orthopaedic Translation. (2024) 49, 62–73, 10.1016/j.jot.2024.09.004, 39430130. PMC11488446 39430130

[51] Zhu S. , Liu H. , Wu Y. , Heng B. C. , Chen P. , Liu H. , and Ouyang H. W. , Wnt and Rho GTPase Signaling in Osteoarthritis Development and Intervention: Implications for Diagnosis and Therapy, Arthritis Research & Therapy. (2013) 15, no. 4, 10.1186/ar4240, 23856044. PMC3979163 23856044

[52] Yang Y. , Zhou H. , Yang J. , Li F. , Hu Q. , Jia Y. , Huang W. , Qin L. , and Zhou Y. , Metformin Attenuates Osteoarthritis Progression by Modulating Mitochondrial Dynamics via Activation of the AMPK/Drp1 Pathway, Journal of Inflammation Research. (2026) 19, 540176, 10.2147/JIR.S540176, 41884158. PMC13012564 41884158

[53] Noda S. , Hosoya T. , Komiya Y. , Tagawa Y. , Endo K. , Komori K. , Koga H. , Takahara Y. , Sugimoto K. , Sekiya I. , Saito T. , Mizoguchi F. , and Yasuda S. , CD34+THY1+ Synovial Fibroblast Subset in Arthritic Joints Has High Osteoblastic and Chondrogenic Potentials in Vitro, Arthritis Research & Therapy. (2022) 24, no. 1, 10.1186/s13075-022-02736-7, 35168627. PMC8845288 35168627

[54] Wang Y. , Zhu L. , Jin S. , Wang Y. , Zeng Z. , Zuo Y. , Xiang X. , Li X. , Huang R. , and Song C. , Identification of Candidate Sex Hormone-Associated Genes and Immune Infiltration Characteristics in Osteoarthritis Based on Bioinformatics Analysis and Machine Learning, PLoS One. (2026) 21, no. 6, e0351556, 10.1371/journal.pone.0351556, 42284285. PMC13262821 42284285

[55] Chen B. Y. , Pathak J. L. , Lin H. Y. , Guo W. Q. , Chen W. J. , Luo G. , Wang L. J. , Sun X. F. , Ding Y. , Li J. , Diekwisch T. G. H. , and Liu C. , Inflammation Triggers Chondrocyte Ferroptosis in TMJOA via HIF-1*α*/TFRC, Journal of Dental Research. (2024) 103, no. 7, 712–722, 10.1177/00220345241242389, 38766865. 38766865

[56] Neurath M. F. and Berg L. J. , VAV1 as a Putative Therapeutic Target in Autoimmune and Chronic Inflammatory Diseases, Trends in Immunology. (2024) 45, no. 8, 580–596, 10.1016/j.it.2024.06.004, 39060140. 39060140

[57] Wegrzyn D. , Zokol J. , and Faissner A. , Vav3-Deficient Astrocytes Enhance the Dendritic Development of Hippocampal Neurons in an Indirect Co-Culture System, Frontiers in Cellular Neuroscience. (2021) 15, 817277, 10.3389/fncel.2021.817277, 35237130. PMC8882586 35237130

[58] Shalom B. , Farago M. , Salaymeh Y. , Sebban S. , Pikarsky E. , and Katzav S. , Vav1 Promotes B-Cell Lymphoma Development, Cells. (2022) 11, no. 6, 10.3390/cells11060949, 35326399. PMC8946024 35326399

[59] Stiffel V. M. , Thomas A. , Rundle C. H. , Sheng M. H.-C. , and Lau K.-H. W. , The EphA4 Signaling Is Anti-Catabolic in Synoviocytes but Pro-Anabolic in Articular Chondrocytes, Calcified Tissue International. (2020) 107, no. 6, 576–592, 10.1007/s00223-020-00747-7, 32816052. PMC7606366 32816052

[60] Zeng R. , Zhuo Z. , Luo Y. , Sha W. , and Chen H. , Rho GTPase Signaling in Rheumatic Diseases, iScience.(2022) 25, no. 1, 103620, 10.1016/j.isci.2021.103620, 35005558. PMC8718819 35005558

[61] Chen J. , Tan J. , Wang N. , Li H. , Cheng W. , Li J. , Wang B. , Sedgwick A. C. , Chen Z. , Chen G. , Zhang P. , Zheng W. , Liu C. , and Chen J. , Specific Macrophage RhoA Targeting CRISPR-Cas9 for Mitigating Osteoclastogenesis-Induced Joint Damage in Inflammatory Arthritis, Cell Reports Medicine. (2025) 6, no. 4, 102046, 10.1016/j.xcrm.2025.102046, 40239628. PMC12047524 40239628

[62] López-Erauskin J. , Bravo-Hernandez M. , Presa M. , Baughn M. W. , Melamed Z. , Beccari M. S. , Agra de Almeida Quadros A. R. , Arnold-Garcia O. , Zuberi A. , Ling K. , Platoshyn O. , Niño-Jara E. , Ndayambaje I. S. , McAlonis-Downes M. , Cabrera L. , Artates J. W. , Ryan J. , Hermann A. , Ravits J. , Bennett C. F. , Jafar-Nejad P. , Rigo F. , Marsala M. , Lutz C. M. , Cleveland D. W. , and Lagier-Tourenne C. , Stathmin-2 Loss Leads to Neurofilament-Dependent Axonal Collapse Driving Motor and Sensory Denervation, Nature Neuroscience. (2024) 27, no. 1, 34–47, 10.1038/s41593-023-01496-0, 37996528. PMC10842032 37996528

[63] Beccari M. S. , Arnold-Garcia O. , Baughn M. W. , Artates J. W. , McAlonis-Downes M. , Lim J. , Leyva-Cázares D. F. , Rubio-Lara H. I. , Ramirez-Rodriguez A. , Bernal-Buenrostro C. N. , Murgia-Bay B. , Rangel C. K. , Kim D. H. , Melamed Z.’. , Lutz C. M. , Lagier-Tourenne C. , Corbett K. D. , López-Erauskin J. , and Cleveland D. W. , Stathmin-2 Enhances Motor Axon Regeneration After Injury Independent of Its Binding To Tubulin, Proceedings of the National Academy of Sciences. (2025) 122, no. 21, e2502294122, 10.1073/pnas.2502294122, 40392845. PMC12130871 40392845

[64] Guerra San Juan I. , Nash L. A. , Smith K. S. , Leyton-Jaimes M. F. , Qian M. , Klim J. R. , Limone F. , Dorr A. B. , Couto A. , Pintacuda G. , Joseph B. J. , Whisenant D. E. , Noble C. , Melnik V. , Potter D. , Holmes A. , Burberry A. , Verhage M. , and Eggan K. , Loss of Mouse Stmn2 Function Causes Motor Neuropathy, Neuron. (2022) 110, no. 10, 1671–1688.e6, 10.1016/j.neuron.2022.02.011, 35294901. PMC9119928 35294901

[65] Shao M. , Wang L. , Zhang Q. , Wang T. , and Wang S. , STMN2 Overexpression Promotes Cell Proliferation and EMT in Pancreatic Cancer Mediated by WNT/*β*-Catenin Signaling, Cancer Gene Therapy. (2023) 30, no. 3, 472–480, 10.1038/s41417-022-00568-w, 36460804. 36460804

[66] Liu G. , He G. , Zhang J. , Zhang Z. , and Wang L. , Identification of SCRG1 as a Potential Therapeutic Target for Human Synovial Inflammation, Frontiers in Immunology. (2022) 13, 893301, 10.3389/fimmu.2022.893301, 35720295. PMC9204521 35720295

[67] Tuerlings M. , van Hoolwerff M. , Houtman E. , Suchiman E. H. E. D. , Lakenberg N. , Mei H. , van der Linden E. , Nelissen R. R. G. H. H. , Ramos Y. Y. F. M. , Coutinho de Almeida R. , and Meulenbelt I. , RNA Sequencing Reveals Interacting Key Determinants of Osteoarthritis Acting in Subchondral Bone and Articular Cartilage: Identification of IL11 and CHADL as Attractive Treatment Targets, Arthritis & Rheumatology. (2021) 73, no. 5, 789–799, 10.1002/art.41600, 33258547. PMC8252798 33258547

[68] Englert M. , Aurbach K. , Becker I. C. , Gerber A. , Heib T. , Wackerbarth L. M. , Kusch C. , Mott K. , Araujo G. H. M. , Baig A. A. , Dütting S. , Knaus U. G. , Stigloher C. , Schulze H. , Nieswandt B. , Pleines I. , and Nagy Z. , Impaired Microtubule Dynamics Contribute to Microthrombocytopenia in RhoB-Deficient Mice, Blood Advances. (2022) 6, no. 17, 5184–5197, 10.1182/bloodadvances.2021006545, 35819450. PMC9631634 35819450

[69] Yue M. , Chen M.-M. , Zhang B. , Wang Y. , Li P. , and Zhao Y. , The Functional Roles of Chemokines and Chemokine Receptors in Colorectal Cancer progression, Biomedicine & Pharmacotherapy. (2024) 170, 116040, 10.1016/j.biopha.2023.116040. 38113624

[70] Burute M. , Jansen K. I. , Mihajlovic M. , Vermonden T. , and Kapitein L. C. , Local Changes in Microtubule Network Mobility Instruct Neuronal Polarization and Axon Specification, Science Advances. (2022) 8, no. 44, eabo2343, 10.1126/sciadv.abo2343, 36332030. PMC9635826 36332030

[71] Kumar A. , Mishra S. , Kumar A. , Raut A. A. , Sato S. , Takaoka A. , and Kumar H. , Essential Role of Rnd1 in Innate Immunity During Viral and Bacterial Infections, Cell Death & Disease. (2022) 13, no. 6, 10.1038/s41419-022-04954-y, 35654795. PMC9161769 35654795

[72] Swaminathan B. , Youn S.-W. , Naiche L. A. , Du J. , Villa S. R. , Metz J. B. , Feng H. , Zhang C. , Kopan R. , Sims P. A. , and Kitajewski J. K. , Endothelial Notch Signaling Directly Regulates the Small GTPase RND1 to Facilitate Notch Suppression of Endothelial Migration, Scientific Reports. (2022) 12, no. 1, 10.1038/s41598-022-05666-1, 35102202. PMC8804000 35102202

[73] Sun Q. , Xu J. , Yuan F. , Liu Y. , Chen Q. , Guo L. , Dong H. , and Liu B. , RND1 Inhibits Epithelial-Mesenchymal Transition and Temozolomide Resistance of Glioblastoma via AKT/GSK3-*β* pathway, Cancer Biology & Therapy. (2024) 25, no. 1, 2321770, 10.1080/15384047.2024.2321770, 38444223. PMC10936657 38444223

[74] Chen Q. , Chen D. , Wang S. , Huang X. , Liang L. , Xie T. , and Lu J. , RND1 Induces Ferroptosis to Alleviate Inflammatory Response, Proliferation, Invasion, and Migration of Rheumatoid Synoviocytes, Journal of Inflammation Research. (2025) 18, 2647–2659, 10.2147/JIR.S500630, 40008082. PMC11853921 40008082

[75] Scotto d’Abusco A. , Cicione C. , Calamia V. , Negri R. , Giordano C. , Grigolo B. , Politi L. , and Scandurra R. , Glucosamine and its N-Acetyl-Phenylalanine Derivative Prevent TNF-Alpha-Induced Transcriptional Activation in Human Chondrocytes, Clinical and Experimental Rheumatology. (2007) 25, no. 6, 847–852, 18173918. 18173918

[76] Voss T.-D. , Hezinger L. , and Kufer T. A. , Pattern Recognition by NOD-Like Receptors, nnate Immunity: Pattern Recognition and Effector Mechanisms. (2025) 1476, 83–105, 10.1007/978-3-031-85340-1_4. 40622540

[77] Zheng C. , The Emerging Roles of NOD-Like Receptors in Antiviral Innate Immune Signaling Pathways, International Journal of Biological Macromolecules. (2021) 169, 407–413, 10.1016/j.ijbiomac.2020.12.127, 33347926. 33347926

[78] Zhang Z. , Huang T. , Chen X. , Chen J. , Yuan H. , Yi N. , Miao C. , Sun R. , and Ni S. , Acetyl Zingerone Inhibits Chondrocyte Pyroptosis and Alleviates Osteoarthritis Progression by Promoting Mitophagy Through the PINK1/Parkin Signaling Pathway, International Immunopharmacology. (2025) 161, 115055, 10.1016/j.intimp.2025.115055, 40494201. 40494201

[79] Sun Y. , Du R. , Shang Y. , Liu C. , Zheng L. , Sun R. , Wang Y. , and Lu G. , Rho GTPase-Activating Protein 35 Suppresses Gastric Cancer Metastasis by Regulating Cytoskeleton Reorganization and Epithelial-to-Mesenchymal Transition, Bioengineered. (2022) 13, no. 6, 14605–14615, 10.1080/21655979.2022.2092677, 35758029. PMC9342288 35758029

[80] Nie L. , Fei C. , Fan Y. , Dang F. , Zhao Z. , Zhu T. , Wu X. , Dai T. , Balasubramanian A. , Pan J. , Hu Y. , Luo H. R. , Wei W. , and Chen J. , Consecutive Palmitoylation and Phosphorylation Orchestrates NLRP3 Membrane Trafficking and Inflammasome Activation, Molecular Cell. (2024) 84, no. 17, 3336–3353.e7, 10.1016/j.molcel.2024.08.001, 39173637. 39173637

[81] Akbal A. , Dernst A. , Lovotti M. , Mangan M. S. J. , McManus R. M. , and Latz E. , How Location and Cellular Signaling Combine to Activate the NLRP3 Inflammasome, Cellular & Molecular Immunology. (2022) 19, no. 11, 1201–1214, 10.1038/s41423-022-00922-w, 36127465. PMC9622870 36127465

[82] Lasser S. A. , Ozbay Kurt F. G. , Arkhypov I. , Utikal J. , and Umansky V. , Myeloid-Derived Suppressor Cells in Cancer and Cancer Therapy, Nature Reviews Clinical Oncology. (2024) 21, no. 2, 147–164, 10.1038/s41571-023-00846-y. 38191922

[83] Li K. , Shi H. , Zhang B. , Ou X. , Ma Q. , Chen Y. , Shu P. , Li D. , and Wang Y. , Myeloid-Derived Suppressor Cells as Immunosuppressive Regulators and Therapeutic Targets in Cancer, Signal Transduction and Targeted Therapy. (2021) 6, no. 1, 10.1038/s41392-021-00670-9, 34620838. PMC8497485 34620838

[84] Rajkumari S. , Singh J. , Agrawal U. , and Agrawal S. , Myeloid-Derived Suppressor Cells in Cancer: Current Knowledge and Future Perspectives, International Immunopharmacology. (2024) 142, Pt A, 112949, 10.1016/j.intimp.2024.112949, 39236460. 39236460

[85] Guo Z. , Chen T. , Wen X. , Fu Q. , Lai J. , Li J. , Chen J. , Weng J. , Wen W. , Cao Q. , Zhu J. , Ye Z. , Yu X. , and Jiang L. , Harnessing the Dual Immunomodulatory Function of Myeloid-Derived Suppressor Cells to Reshape the Inflammatory Microenvironment for Osteoarthritis Therapy, Materials Today Bio. (2025) 35, 102332, 10.1016/j.mtbio.2025.102332, 41080734. PMC12510078 41080734

[86] Akula M. K. , Gilis E. , Hertens P. , Vande Walle L. , Sze M. , Coudenys J. , Incik Y. , Khan O. , Bergo M. O. , Elewaut D. , Wullaert A. , Lamkanfi M. , and van Loo G. , Pyrin Inflammasome-Driven Erosive Arthritis Caused by Unprenylated RHO GTPase Signaling, EMBO Molecular Medicine. (2025) 17, no. 10, 2691–2712, 10.1038/s44321-025-00298-0, 40883609. PMC12514176 40883609

[87] Dusaban S. S. , Chun J. , Rosen H. , Purcell N. H. , and Brown J. H. , Sphingosine 1-Phosphate Receptor 3 and RhoA Signaling Mediate Inflammatory Gene Expression in Astrocytes, Journal of neuroinflammation. (2017) 14, no. 1, 10.1186/s12974-017-0882-x, 28577576. PMC5455202 28577576

[88] Pieren D. K. J. , Benítez-Martínez A. , Descalzo V. , Arando M. , Álvarez-López P. , Garcia-Perez J. N. , Massana N. , Castellón J. , Hoyos-Mallecot Y. , Alvárez-Sierra D. , Cantenys-Molina S. , Ramírez-Serra C. , Rodriguez N. L. , Mañalich-Barrachina L. , Centeno-Mediavilla C. , Castellví J. , Falcó V. , Buzón M. J. , and Genescà M. , Cervical Mucosal Inflammation Expands Functional Polymorphonuclear Myeloid-Derived Suppressor Cells, Mucosal Immunology. (2026) 19, no. 1, 1636–1649, 10.1016/j.mucimm.2025.11.008, 41260389. 41260389

[89] Talbott H. E. , Mascharak S. , Griffin M. , Wan D. C. , and Longaker M. T. , Wound Healing, Fibroblast Heterogeneity, and Fibrosis, Cell Stem Cell. (2022) 29, no. 8, 1161–1180, 10.1016/j.stem.2022.07.006. PMC9357250 35931028

[90] Bensa T. , Tekkela S. , and Rognoni E. , Skin Fibroblast Functional Heterogeneity in Health and Disease, Journal of Pathology. (2023) 260, no. 5, 609–620, 10.1002/path.6159, 37553730. 37553730

[91] Altieri A. , Visser G. V. , and Buechler M. B. , Enter the matrix: Fibroblast-Immune Cell Interactions Shape Extracellular Matrix Deposition in Health and Disease, F1000Research. (2024) 13, 10.12688/f1000research.143506.2, 39886650. PMC11781523 39886650

[92] Peng R. , Lin Q. , Yang Z. , Li H. , Li J. J. , and Xing D. , Fibroblast-Myofibroblast Transition in Osteoarthritis Progression: Current Insights, International Journal of Molecular Sciences. (2025) 26, no. 16, 10.3390/ijms26167881, 40869202. PMC12386718 40869202

[93] Zhao Q. , Shao T. , Zhu Y. , Zong G. , Zhang J. , Tang S. , Lin Y. , Ma H. , Jiang Z. , Xu Y. , Wu X. , and Zhang T. , An MRTF-a-ZEB1-IRF9 Axis Contributes to Fibroblast-Myofibroblast Transition and Renal Fibrosis, Experimental & Molecular Medicine. (2023) 55, no. 5, 987–998, 10.1038/s12276-023-00990-6, 37121967. PMC10238398 37121967

[94] Peterman E. , Murphy A. , Swinburne I. A. , Linbo T. H. , Megason S. G. , and Rasmussen J. P. , Microtubules Regulate Tissue-Level Navigation in Skin-Resident Macrophages, Journal of Cell Science. (2025) 138, no. 18, jcs264101, 10.1242/jcs.264101, 40827096. PMC12516128 40827096

[95] Marcos-Ramiro B. , García-Weber D. , and Millán J. , TNF-Induced Endothelial Barrier Disruption: Beyond Actin and Rho, Thrombosis and Haemostasis. (2014) 112, no. 6, 1088–1102, 10.1160/TH14-04-0299, 25078148. 25078148

[96] Li J. , Zhang W. , Liu X. , Li G. , Gu Y. , Zhang K. , Shen F. , Wu X. , Jiang Y. , Zhang Q. , Zhou F. , Xu K. , and Su J. , Endothelial Stat3 Activation Promotes Osteoarthritis Development, Cell Proliferation. (2023) 56, no. 12, e13518, 10.1111/cpr.13518, 37309689. PMC10693181 37309689

[97] Alexanian M. , Padmanabhan A. , Nishino T. , Travers J. G. , Ye L. , Pelonero A. , Lee C. Y. , Sadagopan N. , Huang Y. , Auclair K. , Zhu A. , An Y. , Ekstrand C. A. , Martinez C. , Teran B. G. , Flanigan W. R. , Kim C. K. S. , Lumbao-Conradson K. , Gardner Z. , Li L. , Costa M. W. , Jain R. , Charo I. , Combes A. J. , Haldar S. M. , Pollard K. S. , Vagnozzi R. J. , McKinsey T. A. , Przytycki P. F. , and Srivastava D. , Chromatin Remodelling Drives Immune Cell-Fibroblast Communication in Heart Failure, Nature. (2024) 635, no. 8038, 434–443, 10.1038/s41586-024-08085-6, 39443808. PMC11698514 39443808

[98] Nanus D. E. , Badoume A. , Wijesinghe S. N. , Halsey A. M. , Hurley P. , Ahmed Z. , Botchu R. , Davis E. T. , Lindsay M. A. , and Jones S. W. , Synovial Tissue From Sites of Joint Pain in Knee Osteoarthritis Patients Exhibits a Differential Phenotype With Distinct Fibroblast Subsets, EBioMedicine. (2021) 72, 103618, 10.1016/j.ebiom.2021.103618, 34628351. PMC8511845 34628351

[99] Tsuchiya M. , Ohashi Y. , Kodera Y. , Satoh M. , Matsui T. , Fukushima K. , Iwase D. , Aikawa J. , Mukai M. , Inoue G. , Takaso M. , and Uchida K. , CD39+CD55- Fb Subset Exhibits Myofibroblast-Like Phenotype and Is Associated With Pain in Osteoarthritis of the Knee, Biomedicines. (2023) 11, no. 11, 10.3390/biomedicines11113047, 38002046. PMC10669511 38002046

[100] Cai S. , Ming B. , Ye C. , Lin S. , Hu P. , Tang J. , Zheng F. , and Dong L. , Similar Transition Processes in Synovial Fibroblasts from Rheumatoid Arthritis and Osteoarthritis: A Single-Cell Study, Journal of Immunology Research. (2019) 2019, 4080735, 10.1155/2019/4080735, 31428656. PMC6681591 31428656

[101] Nobes C. D. , Lauritzen I. , Mattei M. G. , Paris S. , Hall A. , and Chardin P. , A New Member of the Rho family, Rnd1, Promotes Disassembly of Actin Filament Structures and Loss of Cell Adhesion, Journal of Cell Biology. (1998) 141, 187–197, 10.1083/jcb.141.1.187. PMC2132722 9531558

[102] Riou P. , Villalonga P. , and Ridley A. J. , Rnd proteins: Multifunctional Regulators of the Cytoskeleton and Cell Cycle Progression, Bioessays. (2010) 32, no. 11, 986–992, 10.1002/bies.201000060, 20836090. 20836090

[103] D’Urso M. , van den Bersselaar P. , Pragnere S. , Maiuri P. , Bouten C. V. C. , and Kurniawan N. A. , Substrate Stiffness Modulates Phenotype-Dependent Fibroblast Contractility and Migration Independent of TGF-*β* Stimulation, Mechanobiology in Medicine. (2025) 3, no. 4, 100158, 10.1016/j.mbm.2025.100158, 41113512. PMC12529497 41113512

[104] Knights A. J. , Farrell E. C. , Ellis O. M. , Lammlin L. , Junginger L. M. , Rzeczycki P. M. , Bergman R. F. , Pervez R. , Cruz M. , Knight E. , Farmer D. , Samani A. A. , Wu C. L. , Hankenson K. D. , and Maerz T. , Synovial Fibroblasts Assume Distinct Functional Identities and Secrete R-Spondin 2 in Osteoarthritis, Annals of the Rheumatic Diseases. (2023) 82, no. 2, 272–282, 10.1136/ard-2022-222773, 36175067. PMC9972892 36175067

[105] Struglics A. , Okroj M. , Swärd P. , Frobell R. , Saxne T. , Lohmander L. S. , and Blom A. M. , The Complement System Is Activated in Synovial Fluid From Subjects With Knee Injury and From Patients With Osteoarthritis, Arthritis Research & Therapy. (2016) 18, no. 1, 10.1186/s13075-016-1123-x, 27716448. PMC5052889 27716448

[106] Ammitzboll C. G. , Thiel S. , Ellingsen T. , Deleuran B. , Jorgensen A. , Jensenius J. C. , and Stengaard-Pedersen K. , Levels of Lectin Pathway Proteins in Plasma and Synovial Fluid of Rheumatoid Arthritis and Osteoarthritis, Rheumatology International. (2012) 32, no. 5, 1457–1463, 10.1007/s00296-011-1879-x, 21461857. 21461857

[107] Zhou C. , Cui Y. , Yang Y. , Guo D. , Zhang D. , Fan Y. , Li X. , Zou J. , and Xie J. , Runx1 Protects Against the Pathological Progression of Osteoarthritis, Bone Research. (2021) 9, no. 1, 10.1038/s41413-021-00173-x, 34876557. PMC8651727 34876557

[108] Damerau A. , Kirchner M. , Pfeiffenberger M. , Ehlers L. , Do Nguyen D. H. , Mertins P. , Bartek B. , Maleitzke T. , Palmowski Y. , Hardt S. , and Winkler T. , Metabolic Reprogramming of Synovial Fibroblasts in Osteoarthritis by Inhibition of Pathologically Overexpressed Pyruvate Dehydrogenase Kinases, Metabolic Engineering. (2022) 72, 116–132, 10.1016/j.ymben.2022.03.006, 35296430. 35296430

